# Targeting serine/glycine metabolism improves radiotherapy response in non-small cell lung cancer

**DOI:** 10.1038/s41416-023-02553-y

**Published:** 2023-12-30

**Authors:** Anaís Sánchez-Castillo, Elien Heylen, Judith Hounjet, Kim G. Savelkouls, Natasja G. Lieuwes, Rianne Biemans, Ludwig J. Dubois, Kobe Reynders, Kasper M. Rouschop, Rianne D. W. Vaes, Kim De Keersmaecker, Maarten Lambrecht, Lizza E. L. Hendriks, Dirk K. M. De Ruysscher, Marc Vooijs, Kim R. Kampen

**Affiliations:** 1 Department of Radiation Oncology (MAASTRO), GROW School for Oncology and Reproduction, Maastricht University Medical Center+, Maastricht, The Netherlands; 2 Department of Oncology, Laboratory for Disease Mechanisms in Cancer, KU Leuven, and Leuven Cancer Institute (LKI), Herestraat 49, 3000 Leuven, Belgium; 3 Department of Precision Medicine, The M-Lab, GROW School for Oncology and Reproduction, Maastricht University, Maastricht, The Netherlands; 4 Department of Oncology, Experimental Radiation Oncology, KU Leuven, and Leuven Cancer Institute (LKI), Herestraat 49, 3000 Leuven, Belgium; 5 Department of Radiation Oncology, University Hospital Leuven, Leuven, Belgium; 6 Department of Pulmonology, GROW School for Oncology and Reproduction, Maastricht University Medical Center+, Maastricht, The Netherlands

**Keywords:** Cancer therapy, Lung cancer, Cancer metabolism

## Abstract

**Background:**

Lung cancer is the most lethal cancer, and 85% of cases are classified as non-small cell lung cancer (NSCLC). Metabolic rewiring is a cancer hallmark that causes treatment resistance, and lacks insights into serine/glycine pathway adaptations upon radiotherapy.

**Methods:**

We analyzed radiotherapy responses using mass-spectrometry-based metabolomics in NSCLC patient’s plasma and cell lines. Efficacy of serine/glycine conversion inhibitor sertraline with radiotherapy was investigated by proliferation, clonogenic and spheroid assays, and in vivo using a serine/glycine dependent NSCLC mouse model by assessment of tumor growth, metabolite and cytokine levels, and immune signatures.

**Results:**

Serine/glycine pathway metabolites were significantly consumed in response to radiotherapy in NSCLC patients and cell models. Combining sertraline with radiotherapy impaired NSCLC proliferation, clonogenicity and stem cell self-renewal capacity. In vivo, NSCLC tumor growth was reduced solely in the sertraline plus radiotherapy combination treatment group. Tumor weights linked to systemic serine/glycine pathway metabolite levels, and were inhibited in the combination therapy group. Interestingly, combination therapy reshaped the tumor microenvironment via cytokines associated with natural killer cells, supported by eradication of immune checkpoint galectin-1 and elevated granzyme B levels.

**Conclusion:**

Our findings highlight that targeting serine/glycine metabolism using sertraline restricts cancer cell recovery from radiotherapy and provides tumor control through immunomodulation in NSCLC.

## Introduction

Lung cancer is the second most common cancer and remains the leading cause of cancer-related mortality worldwide. Approximately 85% of all lung cancers are classified as non-small cell lung cancer (NSCLC) [1]. NSCLC patients are categorized according to tumor staging, in which stage I represents a small local tumor, and stage IV represents metastatic disease. Depending on, among others, the tumor stage, the current standard of care for patients with NSCLC includes surgical resection, radiotherapy (RT), chemotherapy, immunotherapy, targeted therapies, or combinations of these modalities. In the curative treatment setting, patients with inoperable stage I and II NSCLC, due to comorbidities, are treated with stereotactic RT, with local control rates of >90% [2]. In locally advanced (stage III) NSCLC, most fit patients receive concurrent chemotherapy with a platinum-doublet and RT (CCRT), followed by one year of durvalumab (anti-PD-L1) therapy [3]. Especially in the metastatic setting, an increasing number of targeted therapies either in first line or beyond, has become available for oncogenic drivers such as KRAS G12C, EGFR, ALK, ROS1, BRAF V600, MET, RET and NTRK. Standard of care treatment for patients without an option for first line targeted therapy is a PD-(L)1 inhibitor, either as monotherapy or combined with chemotherapy with or without a CTLA-4 inhibitor. Even with the recent improvements due to (adjuvant) immunotherapy treatment, collectively, more than 50% of patients with NSCLC still die of lung cancer within 5 years [1, 4]. This indicates a strong need for better understanding of the tumor response to the treatment to overcome treatment resistance and reduce tumor progression, as well as to identify therapeutic selectivity, increasing the treatment sensitivity of the tumor while reducing adverse effects in normal tissue [1]. Metabolic reprogramming is a hallmark of cancer cells and metabolic vulnerabilities of cancer cells are promising targets to be exploited to improve the therapeutic efficacy and cure rates. Many of the genetic defects in NSCLC trigger metabolic rewiring towards glycolysis. Interestingly, approximately 37% of adenocarcinoma lung cancer cases present overexpression of the enzymes of the de novo serine/glycine (ser/gly) synthesis pathway, which branches from the glycolysis via the glycolytic intermediate 3-phosphoglycerate (3-PG) [5]. The activation of de novo ser/gly synthesis pathway has been observed, both because of serine synthesis gene amplifications and alterations of upstream regulators that promote enhanced enzyme overexpression. For instance, KRAS mutations drive cancer cell metabolic dependency by increasing glycolysis and subsequently feeding the ser/gly synthesis side branch via NRF2 upregulation, which is a transcription factor that is frequently deregulated in NSCLC [6]. LKB1 phosphorylates and activates AMP-activated protein kinase (AMPK), which functions as an energetic sensor, regulating glucose and lipid metabolism in response to alterations in nutrients and intracellular energy levels [7]. Moreover, LKB1 loss has been linked to increased serine metabolism [8]. BRAF mutated cancers also present elevated glycolysis and increased serine synthesis flux [9, 10]. Strikingly, upregulation of the de novo ser/gly synthesis pathway has been shown to cause resistance to BRAF inhibitors in NSCLC cells [11].

The de novo synthesis of serine from 3-PG occurs in a chain of consecutive enzymatic reactions catalyzed by phosphoglycerate dehydrogenase (PHGDH), phosphoserine aminotransferase 1 (PSAT1) and phosphoserine phosphatase (PSPH). The reversible interconversion of serine and glycine is catalyzed by, either the cytosolic or mitochondrial serine hydroxymethyltransferase (SHMT), SHMT1 or SHMT2, respectively, of which the latter is most prominently expressed in cancer [12–15]. This reaction donates carbon units to the one-carbon metabolism, comprised of folate and methionine cycles. The trans-sulfuration pathway is also connected to the methionine cycle through the intermediate homocysteine. Serine condenses with homocysteine to generate cystathionine, which is then cleaved to produce α-ketoglutarate (α-KG) and cysteine. In addition, both cysteine and glycine are essential players in controlling redox balance through the synthesis of the antioxidant glutathione (GSH). Importantly, de novo ser/gly synthesis pathway activation in lung cancer cells leads to increased GSH synthesis and folate-dependent NADPH production to regulate redox homeostasis, and enhanced purine synthesis to sustain proliferation [16–18]. Consequently, ser/gly metabolism has been linked to tumor pathogenesis, poor outcome, and resistance to chemotherapy and targeted therapies in NSCLC [5, 16, 17].

The role of ser/gly metabolism in response to RT treatment remains largely unknown. We propose an important contribution of de novo ser/gly synthesis pathway activation to RT responses by supplying ATP, nucleotides, and antioxidants to support the survival and/or recovery of cancer cells upon RT. Therefore, current approaches that target the de novo ser/gly synthesis pathway might be a promising strategy for therapeutic intervention in combination with RT [5, 19]. Our lab recently identified sertraline as a potent SHMT inhibitor. Sertraline is a selective serotonin reuptake inhibitor (SSRI), which is a widely used anti-depressant in the clinic, and consequently its safety and pharmacokinetic properties have already been assessed [20]. Sertraline selectively inhibited the proliferation of serine synthesis dependent breast cancer, RPL10 R98S T-cell acute lymphoblastic leukemia (T-ALL) cells, and NKX2-1 T-ALL and NSCLC cells. The therapeutic dosages for patients receiving sertraline as antidepressant vary between 50 and 250 mg/day, which are well tolerated and with minimal impact on the main cytochrome P450 enzymes. Therefore, in the case of sertraline, limited clinically significant drug interactions have been reported, and to a lesser extent than for other SSRIs [20, 21]. Furthermore, sertraline has low affinity towards cholinergic, histaminergic, and noradrenergic receptors, causing minor cardiovascular and sedative effects [22]. Our previously used concentrations of sertraline in pre-clinical in vivo experiments with anti-cancer effects can be extrapolated to the concentrations used in humans, showing a therapeutic index of sertraline that could support future clinical trials [19, 23, 24].

Here, we investigated the use of sertraline as SHMT inhibitor in combination with RT. In vitro, we found strong synergistic anti-cancer effects on proliferation and clonogenic capacity, as well as impairment of long-term intrinsic repopulating frequency and self-renewal of NSCLC stem-like cells. In vivo, we observed significantly improved tumor control using RT in combination with sertraline.

Besides cancer cell intrinsic metabolic rewiring, the tumor microenvironment can be extrinsically influenced by cancer cell-derived metabolites [24, 25]. Our group has recently published findings indicating that isogenic NKX2-1-driven ser/gly synthesis dependent lung tumors display enhanced aggressiveness in vivo, which is associated with systemic metabolic reprogramming, as indicated by the increase in the ser/gly metabolism-derived metabolite, i.e., GSH, accompanied by a decrease in L-cysteine, one of the building blocks of GSH, into the bloodstream [23]. Therefore, we aimed to study systemic metabolic effects, as well as local metabolic effects, of ser/gly producing NSCLC in response to RT in combination with sertraline. Interestingly, analysis of blood plasma samples from patients with NSCLC suggested that ser/gly pathway metabolites are associated with suppression of the immune system. In vivo, we observed strong associations between systemic tumor-derived ser/gly pathway metabolites and tumor weights, and the combination of sertraline and RT improved the anti-tumor immune landscape in vivo.

## Materials & methods

### Stage I and III NSCLC patient plasma samples

Patients diagnosed with stage I or stage III NSCLC scheduled for curative-intent RT and chemoradiation (CCRT), respectively, were prospectively included at MAASTRO (Maastricht, The Netherlands) and University Hospitals Leuven (UZ Leuven, Leuven, Belgium) between May 2017 and March 2019. This study was approved by the ethics committees of UZ/ KU Leuven and MUMC (NL59321.068.16/ METC 163073). Patient samples were obtained after a signed informed consent in agreement with Helsinki’s declaration. This study was registered in ClinicalTrials.gov Identifier: (NCT02921854) [26].

### Targeted metabolomics mass spectrometry

Metabolomics mass spectrometry was outsourced to the VIB metabolomics expertise center, Leuven, Belgium. Samples were processed as follows, 10 µl of blood plasma was added to 990 µl ice-cold mystric acid containing 80% methanol-based extraction buffer, stored at −80 °C for one day, centrifuged and sent for targeted identification of metabolite levels by mass spectrometry analysis. Relative abundances of serine and glycine pathway metabolites were measured and normalized to the quality control runs of those targets. Mass spectrometry was performed using a Q Exactive Focus hybrid quadrupole-Orbitrap. Peak identification was performed by the core facility. Repeats were performed to assure reproducibility of NSCLC patient serum samples (Pearson’s correlation r = 0.935, *p* = 2,48E−46 for serine and r = 0.878 *p* = 2,1422E−33 for threonine, Supplementary Fig. S1). A similar approach was applied to measure metabolite levels in mouse tumor fluids and serum.

### Cell lines

NSCLC cell lines, NCI-H1299 and Calu-6, and Lewis lung carcinoma cells (LLC, passage #3) were cultured at 37 °C and 5% CO_2_ in DMEM (Sigma-Aldrich) supplemented with 10% fetal bovine serum (Sigma-Aldrich). NSCLC cell lines, NCI-H520, HCC15, NCI-H125 and NCI-H1975, were cultured at 37 °C and 5% CO_2_ in RPMI-1640 (Sigma-Aldrich) supplemented with 10% fetal bovine serum. NCI-H1299, Calu-6, NCI-H520 and NCI-H1975 cell lines were obtained from ATCC, HCC15 was obtained from Leibniz-Institute DSMZ and NCI-H125 was received from Molecular Cell Biology, University of Maastricht. LLC cells were cultured in serine–glycine-glucose-free RPMI-1640 (Teknova) supplemented with 2 mg/mL glucose (Sigma-Aldrich) and with 10% dialyzed serum (Life Technologies, A3382001). Primary bronchial epithelial cells (PBEC) cells were kindly provided by the Primary lung culture (PLUC) facility at MUMC+, Maastricht, The Netherlands. Healthy lung tissue used for the isolation of PBECs was obtained from the Maastricht Pathology Tissue Collection (MPTC) and originated from tissue resected during lobectomies of patients who underwent surgery for lung cancer. Collection, storage, and use of tissue and patient data were performed in agreement with the Code for Proper Secondary Use of Human Tissue in the Netherlands (http://www.fmwv.nl). The scientific board of the MPTC approved the use of materials for the present study under MPTC2010-019. In addition, formal permission was obtained from the local Medical Ethical Committee (METC) code 2017-0087 and patients have provided written consent to the use of the material for research. PBEC cells were cultured in Keratinocyte Serum Free Medium (KSFM) (Invitrogen) supplemented with 1 mM isoproterenol (Sigma-Aldrich), mycozap (Sigma), 2.5 ng Human EGF recombinant protein (ThermoFisher), and bovine pituitary extract (ThermoFisher) in fibronectin (Corning) coated dishes. LLC cells were tested by QM Diagnostics and were found negative for MAP17. All cell lines were regularly tested for *Mycoplasma* contamination.

### ^13^C_6_-glucose tracing metabolomics

NCI-H1299, Calu-6, NCI-H520, NCI-H125 and NCI-H1975 cells were plated in 3 mL of respective media in 6-well plates. PBEC cells were used as healthy lung cells from two different donors, i.e., donor 127 and donor 161. After 24 h of incubation at 37 °C, NCI-H1299, Calu-6, NCI-H520, NCI-H125 and NCI-H1975 and PBEC cells were washed with PBS, and the tracing medium was added, i.e., glucose-free DMEM or glucose-free RPMI-1640 with 10% dialyzed serum (Gibco) and 4.5 g/L ^13^C_6_-glucose (Sigma) or 2 g/L^13^C_6_-glucose, respectively. After one hour incubation at 37 °C, NCI-H1299, Calu-6, NCI-H520, NCI-H125 and NCI-H1975 and PBEC cells were irradiated with a single dose of 6 Gy using a MCN 225 X-ray tube (Phillips) operated at 225 keV and 10 mA and incubated at 37 °C for 24 h. For the preparation of media samples for metabolomics, 10 µl of media was collected and added 990 µl of medium extraction buffer (80% methanol, containing 2 µM d27 myristic acid). The samples were centrifuged for 15 min at 4 °C using 20.000 × *g* and 250 µl was transferred to a new tube. For preparation of cell extracts for metabolomics, cells were washed with ice cold (4 °C) 0.9% NaCl solution. Subsequently, 300 µl of cellular extraction buffer (80% methanol, containing 2 µM d27 myristic acid) was added to the cells and incubated 2-3 min on ice before scraping the cells. The samples were centrifuged at 20.000 × *g* for 15 min at 4 °C. The protein pellet was analyzed by Pierce BCA Protein Assay kit (Thermofisher) to determine the protein concentrations and the supernatant was transferred to a new tube for the identification of metabolite levels by mass spectrometry analysis at the Metabolomics Expertise Center (MEC - KU Leuven/VIB).

### Real-time confluency imaging

NCI-H1299, Calu-6, NCI-H520, HCC15, NCI-H125 and NCI-H1975 (150 μl) were seeded at seeding densities between 1000–3000 cells/well in 96-well plates (Greiner CELLSTAR, Sigma-Aldrich) and incubated at 37 °C overnight. Next, 50 μl of 4x compound solutions, i.e., DMSO as control and sertraline (Sigma-Aldrich), diluted in the respective media with 10% FBS, were added, reaching a final DMSO concentration of 0.1%/well. The final concentrations of sertraline were 10 µM for NCI-H1299, NCI-H520, HCC15, NCI-H125 and NCI-H1975 and 5 µM for Calu-6 cell line. After 48 h of treatment with sertraline, cells were irradiated with single doses of 0 and 6 Gy using a MCN 225 X-ray tube (Phillips) operated at 225 keV and 10 mA. Treatment schedule is based on our previous observations of sertraline effects only after 48 h treatment [19]. Cell proliferation was assessed by real-time imaging of confluency on an IncuCyte Zoom system (Essen BioScience) with 6 technical replicates per condition. The area under the curve (AUC) was calculated using GraphPad Prism (v.9.2.0) and fold change difference was calculated between AUC of DMSO and the different treatments. Next, we used the bliss independence model, in which the drug effects from single and combination treatments are expressed in the form of a probability (0 ≤ E ≤ 1), and determined the calculation index (CI) using the following formula:

$${CI}=\frac{{Ea}+{Eb}-{EaEb}}{{Eab}}$$

where ${Ea}$ is 1 minus effect of RT treatment, ${Eb}$ is 1 minus the effect of sertraline treatment and ${Eab}$ is 1 minus the effect of the combination treatment. CI will be indicative of synergy, antagonism or additivity when CI is under, above or equal to 1, respectively [27].

### Clonogenic survival assay

NCI-H1299 (100,000 cells), Calu-6 (300,000 cells), NCI-H520 (400,000 cells), NCI-H125 (150,000 cells) and NCI-H1975 (150,000 cells) and HCC15 (150,000 cells) were seeded in 60 mm cell culture plates and incubated at 37 °C overnight. Next, cells were treated with DMSO and sertraline for 48 h, reaching a DMSO concentration of 0.1%. The final concentrations of sertraline were 10 µM for NCI-H1299, 7 µM for NCI-H520, HCC15, NCI-H125 and NCI-H1975 and 2.5 µM for Calu-6 cell line. These concentrations of sertraline were based on reaching ~50% reduction in clonogenic survival when used as monotherapy. To investigate the effect of sertraline in combination with RT, cells were irradiated with single doses of 0, 2, 4 and 6 Gy after 48 h pre-treatment with sertraline, using a MCN 225 X-ray tube (Phillips) operated at 225 keV and 10 mA, in the case of NCI-H125 cell line, and using a clinical linear accelerator (LINAC), 10 MV FF beams at a dose rate of 4 Gy/min, for NCI-H1299, NCI-H520, NCI-H1975, NCI-HCC15 and Calu-6 [28]. Cells were de-attached immediately after RT and plated as single cells in 60 mm plates at the optimized cellular density to obtain ≥ 20 colonies at the end of the assay. Sertraline was re-added for 7 days. Next, sertraline was removed, and colonies were allowed to form with new media at 37 °C in 5% CO_2_ for another 7–10 days. Cells were fixed and stained with 70% ethanol, 0.4% (w/v) methylene blue and destained with distilled water. Colonies with >50 cells were counted. The surviving fractions after the respective radiation dose are presented as a fraction of the growth of untreated colonies. Survival curves are represented using a linear quadratic model, least squares regression with weighting by 1/Y^2, and extra sum-of-squares F test, comparing A and B parameters, to evaluate significance.

### Colony replating assay

After performing the clonogenic assay as indicated above, cells in the colonies (*n* = 3 plates for each condition) were de-attached, and the viable cells were counted, showing similar and representative reductions on absolute viable cell counts (GUAVA easycyte) as previously observed in the number of colonies (Supplementary Fig. S2). For the replating assay, 500 viable cells/well were plated in a 60 mm dish and allowed to form colonies again for 10 days. After 10 days, cells were fixed and stained with 70% ethanol, 0.4% (w/v) methylene blue and detained with distilled water. Colonies with >50 cells were counted.

### Limiting dilution spheroids assay

A clonogenic survival assay of Calu-6 cells was performed as described above. After allowing cells to form colonies for 17 days, colonies were de-attached. The viable cells were counted (GUAVA easycyte) and 1, 5, 10, 25 and 50 cells were seeded in 10 technical replicates in 96-well clear round bottom ultra-low attachment plates (Corning). Cells were seeded in advanced DMEM/F-12 (Thermofisher) supplemented with R-Spondin (10% v/v) and Noggin (10% v/v) conditioned media, B-27 supplement (Thermofisher) (1X), nicotinamide (10 mM) and FBS (1%). Cells were allowed to form spheres for 10 days, without re-adding any treatment. After 10 days, the number of wells containing spheres was determined for each condition and the software ELDA (Extreme limiting dilution analysis) was used to calculate the long-term cancer stem cell initiating frequency and significance [4]. This assay was repeated three times (0–30 days) and the fold changes of the confidence interval estimate values were calculated, using the non-irradiated cells without treatment as controls.

### Cytoplasmatic ROS levels

Cells were seeded and allowed to attach overnight. Next, cells were treated with sertraline for 48 h and subsequently irradiated. After 24 h, ROS levels were analyzed by incubating NSCLC cells with 2,5 µM of CellROX Green Reagent (Thermofisher) in serum free medium at 37 °C for 30 min. The analysis was performed in FACS Canto II cytometer and BD FACS Diva 6.1.1 software was used. FlowJo V10.8 was used to: exclude doublets and cellular debris and to analyze the mean fluorescent intensity (MFI). MFI was normalized to the DMSO control to obtain the Fold-Change.

### In vivo tumor growth

The animal experiment was performed with appropriate ethical approval (Maastricht University, AVD1070020185464). C57BL/6 mice were injected in the right flank with 1.0 ×10^6^ LLC cells. Mice were randomized in different treatment groups: control (*n* = 4), RT (*n* = 3), sertraline (*n* = 4) and the combination of sertraline and RT (*n* = 4). Tumors were locally irradiated with 15 Gy using a clinical linear accelerator (LINAC), 15 MeV, 3 Gy/min, SSD 100 cm, on day 10 when the tumors reached a volume of 200 mm^3^. All groups received intraperitoneal treatment injections, either DMSO (10%) or sertraline (15 mg/kg) diluted in PBS up to a volume of 500 µl. Sertraline treatment was initiated prior RT, i.e., on day 7, and repeated every 3 days until animal killing on day 17. At the end of the study, tumors were rapidly excised, weighed, and flash-frozen in liquid nitrogen for further immunoblot analysis. Animals prematurely reaching a human endpoint were discarded from tumor weight analysis, leaving *n* = 3 controls, *n* = 3 RT, *n* = 3 sertraline, *n* = 4 combination therapy. After mechanical dissociation of the tumors in RPMI media, tumor cells were used for flow cytometry analysis and the supernatant with tumor fluids was collected and stored at −80 °C for metabolomics and cytokine analysis. Terminal blood was collected via cardiac puncture. Blood samples were allowed to clot for 2 h at room temperature and centrifuged for 20 min at 2000 × *g*. Following, serum was removed and stored at −80 °C for metabolomics.

### Flow cytometry analysis

SHMT2 expression upon irradiation was analyzed in NSCLC cell lines using the antibody SHMT2 E7X5B (Cell Signaling, 93566S). Cell-bound IFN-γ, CD11b, CD8 and CD4 expression in LLC single tumor cells upon the different treatments was analyzed by flow cytometry using the antibodies: PE/Cy7 anti-mouse IFN-y (Biolegend, 505825), PE-Cy7 Rat Anti-Mouse CD11b (BD Pharmingen, 552850), V500 Rat anti-mouse CD8a, clone 53–6.7 (BD Horizon, 560776), FITC conjugated anti-mouse CD4, clone RM4–5 (Ebioscience, 11-0042-85) according to manufacturer’s instructions. The analysis was performed on a FACS Canto II cytometer and BD FACS Diva 6.1.1 software was used. FlowJo V10.8 was used to analyze the MFI of SHMT2 and the % of cell-bound IFN-γ, % of CD11b+, % of CD8+ and % of CD4+ cells.

### Immunoblot

Cells were lysed in protein lysis buffer (Cell Signaling) supplemented with Roche complete™ Protease Inhibitor Cocktail tablets (Sigma-aldrich). Tumor samples were mechanically dissociated in protein lysis buffer in proportional volumes to the weight of the tumor pieces. Proteins were boiled with laemmli sample buffer (Biorad) plus 2-mercaptoethanol as a loading buffer. Equal amounts of extracts were loaded in 4–15% Criterion TGX Precast Midi Protein gels (Biorad). Proteins were transferred to PVDF membranes (Thermofisher) using a Power Blotter–Semi-dry Transfer System (Thermofisher) and incubated overnight with IFN-γ (E3V1X) Rabbit mAb (Cell Signaling, #98139S), Galectin-1/LGALS1 (D608T) Rabbit mAb (Cell Signaling, #12936S, Granzyme B (E5V2L) Rabbit mAb (Cell signaling, #44153) and SHMT2 (E7F4Q) Rabbit mAb (Cell signaling, #3344), vinculin Mouse mAb (Sigma-aldrich, #V9131), actin (clone C4) Mouse mAb (MP biomedicals, #691001) antibodies diluted in 5% skimmed milk in Tris-Buffered NaCl Solution with Tween 20 (TBST). After washing with TBST, membranes were incubated for 1 h at room temperature with the secondary antibodies conjugated to horseradish peroxidase (HRP) anti-rabbit IgG (Cell Signaling) or anti-mouse IgG (Cell Signaling). ECL reagents (Sigma-Aldrich) were used to visualize the proteins in Azure C600 immunoblot imager. Image Studio Lite was used for the quantitative analysis of protein expression.

### Cytokine array

A cytokine array was performed on the LLC tumor fluids (*n* = 3 per treatment arm) using the proteome profiler mouse XL cytokine array (R&D) following the manufacturer’s instructions. Briefly, capture antibodies were spotted in duplicate on nitrocellulose membranes to bind specific target proteins. The captured proteins were then detected with biotinylated detection antibodies and visualized using chemiluminescent detection reagents. Signaling intensity of the cytokines was analyzed with Image Studio Lite. Data normalization was achieved by determining the mean signal over each array. Cytokine spot intensities from duplicates were divided by the mean array signal. Sample randomization was applied by using one array kit (4 membranes for 4 samples) for one sample per treatment group.

### Statistics

The number of biological replicates per experiment and the number of experiments performed for each data set and the statistical analysis performed are stated in the figure legends. Results are depicted as mean ± standard deviation (SD) unless otherwise stated. Statistical analyses were performed using GraphPad Prism (v.9.2.0) or IBM SPSS software based upon F-test for Equality of Variances unless otherwise stated. The box plots present five sample statistics: the minimum, the lower quartile, the median, the upper quartile, and the maximum. Whiskers/inner fences are defined based upon the distribution of the data points, up to a maximum of 1.5 times the height of the box. Outliers are presented in each plot, when exceeding the whiskers. Rounded outliers lay in the range of 1.5–3 times the height of the box and asterisks or stars are extreme outliers that have values more than three times the height of the boxes. For in vitro experiments, comparative analyses between different experimental groups were performed using t-student test and one-way ANOVA with Tukey post hoc tests for intergroup comparisons unless otherwise stated. Non-parametric Kruskal–Wallis test with Dunn’s multiple comparisons test was used to test RT response on patient’s metabolite plasma levels and the effect of the combination of sertraline and RT on tumor weight in the mouse model. Based upon tumor weight effects, we used the Jonckheere-Terpstra test to define the effect of combination therapy over DMSO and monotherapies. The analysis of associations between metabolites and immune signatures in blood plasma samples of patients with NSCLC was indicated using Pearson correlation coefficients. For the cytokine array, the data was normalized and therefore, we used Welch’s ANOVA test with Dunnett’s T3 multiple comparisons test. Results were considered significant if the p-value was <0.05 (*), <0.01 (**), <0.001 (***), or <0.0001 (****).

## Results

### Plasma from patients with NSCLC displays decreased levels of serine synthesis-derived glutathione and its precursor homocysteine in response to RT

**Fig. 1:**
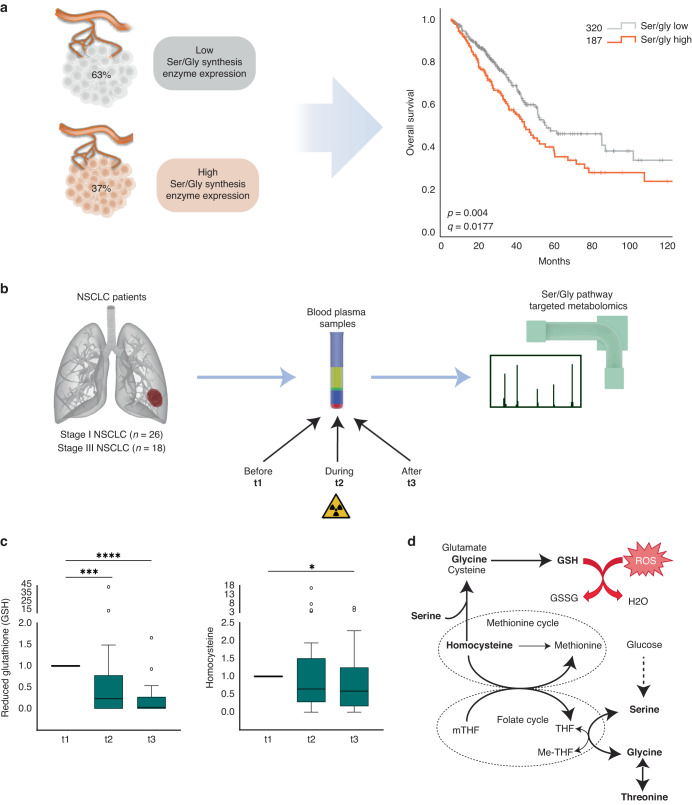
Homocysteine and glutathione (GSH) levels decrease in response to RT in NSCLC patient’s plasma samples. **a** Lung adenocarcinoma (TCGA PanCancer Atlas) dataset collected in cBioportal was used to identify the % of lung adenocarcinoma cancer patients with high and low expression of ser/gly synthesis enzymes, i.e. *PHGDH*, *PSAT1*, *PSPH*, *SHMT1/2* using the standard setting in cBioportal (genetic alterations e.g. amplifications + RNA sequencing z-score threshold = 2.0 relative to diploid samples). The Kaplan Meier curve compared the overall survival of patients with with high versus low ser/gly synthesis enzyme expression. **b** Study design of plasma metabolite analysis. Patient blood samples of stage I/III NSCLC patients were collected before (t1), during (t2), and after chemo-radiation therapy (t3). Stage I NSCLC patients received stereotactic radiotherapy and stage III NSCLC patients received concurrent chemo-radiation therapy. Blood plasma was extracted and processed for mass spectrometry analysis using a LC-Q-Exactive Focus orbitrap. Ser/gly synthesis pathway metabolites were analyzed. **c** Box plots showing homocysteine and gluthatione (GSH) plasma levels during (t2) and after RT (t3) relative to the values before RT (t1). Kruskal–Wallis test with a Dunn’s multiple comparisons test was performed, where *p-value  <  0.05, ***p-value < 0.001, ****p-value < 0.0001. **d** Serine and glycine bidirectional inter-conversion donates carbon units to one carbon metabolism, comprised by folate and methionine cycle, and therefore being upstream metabolites of homocysteine and GSH synthesis. Homocysteine and GSH are important during and after RT in patients with NSCLC.

Many cancer cells acquire dependency on de novo production of serine and glycine for continuous cell growth, proliferation and survival [5, 15]. As previously noted, NSCLC patient tumor samples showed higher expression of the different enzymes of the de novo ser/gly synthesis pathway, i.e. *PHGDH*, *PSAT1*, *PSPH* and *SHMT2*, compared to the adjacent normal lung tissue, which hardly showed any expression of ser/gly pathway enzymes. Interestingly, the expression of these genes increases with tumor staging of NSCLC (Supplementary Fig. S3, *p* < 0.0001 for *PSAT1, PSPH* and *SHMT2*). In line with this observation, the 37% of lung adenocarcinoma cases that present elevated expression of ser/gly synthesis enzyme genes displayed worse overall survival rates as compared to the 63% of lung adenocarcinoma cases with low expression of ser/gly synthesis enzymes genes (*n* = 187 and *n* = 320, respectively, *p* = 0.004*, q* = 0.0177) (Fig. 1a). Hence, these data suggest an important contribution of ser/gly metabolism in the outcome of patients with NSCLC. Yet, the role of ser/gly metabolism in the survival and recovery of NSCLC from the current standard-of-care treatments, such as RT, still needs to be addressed.

Since we previously found that ser/gly rewired tumors produce and secrete high levels of pathway metabolites into the circulation, we hypothesized that metabolomic analysis of blood plasma samples from patients with NSCLC could give valuable insight into ser/gly metabolic rewiring in tumor cells in response to RT [24]. A set of 37 blood plasma samples from patients with stage I/III NSCLC receiving stereotactic RT (stage I) or concurrent chemo-radiation (CCRT, stage III) were previously collected at three different time points: before (t1), during (t2), and after (t3) the last dose of radiation (Fig. 1b) [26]. We performed targeted mass spectrometry–based metabolomics of these plasma samples and discovered that blood plasma levels of the antioxidant GSH were significantly reduced after the first dose of the treatment, and even further decreased after the last dose (Fig. 1c, p = 0.0002 and *p* < 0.0001). The decrease in GSH might be attributed to either reduced GSH production or secretion of GSH into the bloodstream. In situations of oxidative stress, such as radiation-induced oxidative stress in tumor cells, the antioxidant GSH will be oxidized into GSSG, resulting in depletion of GSH and subsequently less secretion into the bloodstream. In line with the reduced GSH levels, we observed decreased levels of the upstream metabolite homocysteine (Fig. 1c, *p* < 0.05). Homocysteine is a precursor of cysteine, which in combination with glycine and glutamate generates GSH. Collectively, NSCLC plasma samples showed a reduction in homocysteine/GSH levels following RT treatment, suggesting increased usage of these metabolites by NSCLC cells in response to RT (Fig. 1d).

### Ser/gly pathway metabolites are required for RT recovery in NSCLC

**Fig. 2:**
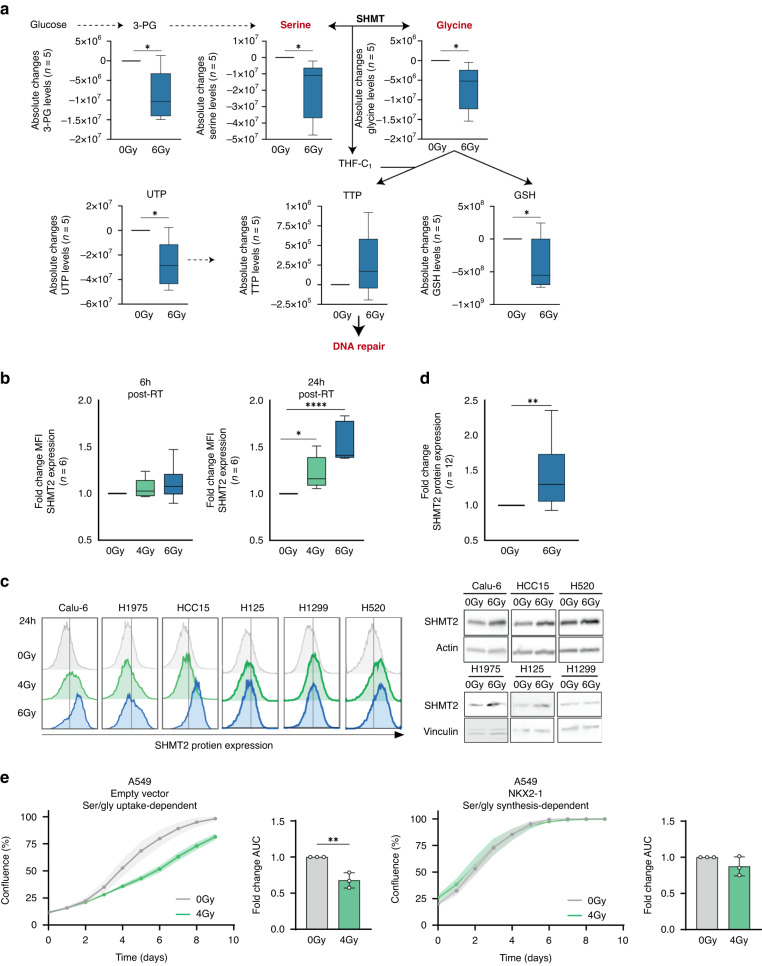
Serine and glycine intracellular levels decrease in response to irradiation in NSCLC cell lines. **a** Box plots showing the intracellular levels of 3-PG, serine, glycine, GSH, UTP and TTP in in a pannel of 5 NSCLC cell lines 24 h after 6 Gy irradiation. Unpaired 2-tailed t-test was used. **b** Fold change of mean fluorescence intensity (MFI) of SHMT2 protein expression using flow cytometry in NSCLC cell lines (*n* = 6) in response to irradiation, 4 Gy and 6 Gy for 6 and 24 h. An unpaired 2-tailed t test was used to compare irradiated cells vs control cells. **c** Flow cytometry graphs of SHMT2 protein expression in NSCLC and cell lines in response to irradiation, 4 Gy and 6 Gy for 24 h. **d** Fold change of SHMT2 protein expression measured by immunoblot in *n* = 6 NSCLC cell lines 24 h post-irradiation and *n* = 2 biological replicate for each cell line. An unpaired 2-tailed t test has been used to compare irradiated cells and control cells. **e** Cell growth curves showing the confluence (%) of A549 control cells and A549 cells with overexpression of NKX2-1 upon exposure to 4 Gy irradiation. Fold change of area under the curve (AUC) of the A549 control NKX2-1 overexpression cell growth curves. Each dot represents an independent experiment. Unpaired 2-tailed t-test has been performed. Statistical analysis *p-value < 0.05, **p-value < 0.01, ****p-value < 0.0001.

To validate the observations in the plasma of patients with NSCLC and confirm our hypothesis of augmented ser/gly pathway dependency, we performed ^13^C_6_-glucose tracing and metabolomics analysis in a panel of 5 NSCLC cell lines, i.e., NCI-H1299, NCI-H125, NCI-H1975, Calu-6 and NCI-H520 to identify the RT related metabolic changes. Firstly, we observed that NSCLC cell lines with the highest glucose-derived serine and glycine, and therefore the highest baseline de novo ser/gly synthesis, were more radioresistant, i.e., NCI-H1299 and NCI-H125, compared to the NSCLC cell lines with lower baseline de novo ser/gly synthesis, e.g., NCI-H520 (Supplementary Fig. S4A, B). Furthermore, the most radioresistant NSCLC cell model NCI-H1299 showed elevated ser/gly production, reflected as the accumulation of labeled m + 3 serine and m + 2 glycine, in response to irradiation (Supplementary Fig. S4C). While the other NSCLC cell lines (n = 4) did not show changes in the labeled glucose used for ser/gly production (Supplementary Fig. S4D), we observed a strong collective decrease in the intracellular levels of the glycolytic intermediate 3-PG in response to 6 Gy irradiation (*p* = 0.014), as well as downstream intracellular levels of serine and glycine metabolites (*p* = 0.041 and *p* = 0.025, respectively, Fig. 2a), while ser/gly metabolites in the media remained stable (Supplementary Fig. S4E). In addition, serine and glycine metabolism is involved in the synthesis of the antioxidant GSH, which was decreased in response to irradiation (*p* = 0.031) (Fig. 2a).

Serine and glycine are the major sources of carbon units to the one-carbon metabolism, being involved in the synthesis of nucleotides [16, 29]. Cytidine triphosphate (CTP), adenosine triphosphate (ATP) and guanosine triphosphate (GTP) nucleotides were reduced (*p* = 0.035, *p* = 0.020, *p* = 0.018, respectively) (Fig. 2a, Supplementary Fig. S4F). Interestingly, we observed a trend of increased levels of the pyrimidine nucleotide thymidine triphosphate (TTP) in irradiated cells (*p* = 0.211), while irradiation led to a significant decrease of the cellular levels of uridine triphosphate (UTP) (*p* = 0.012), the precursor nucleotide of TTP. Serine catabolism initiated by SHMT1/2 transfers one-carbon unit to tetrahydrofolate (THF) forming glycine and 5,10-methylene-THF (me-THF). Me-THF functions as one-carbon donor, supporting the methylation reaction of uridine monophosphate (UMP) to generate thymidine monophosphate (TMP). The supply and maintenance of a balanced pool of deoxythymidine triphosphate (dTTP) nucleotide is essential for adequate DNA replication and repair. For instance, an increased dUTP/dTTP ratio leads to misincorporation of uracil during DNA replication [30, 31]. In addition, dTTP pools facilitate DNA repair under genotoxic stress [32]. Our observations show increased consumption of ser/gly pathway metabolites in order to facilitate dTTP production, which may promote DNA repair and aid in recovery of NSCLC cells in response to irradiation, as NSCLC specific response. In line with the previously shown lack of enzyme expression in “normal” lung tissue (Supplementary Fig. S3), “normal” adjacent lung cells from patients with lung cancer did not show this enhanced ser/gly metabolite consumption, synthesis, or changes in the media upon irradiation ex vivo (Supplementary Fig. S4A, G, H). These results collectively indicate that ser/gly pathway metabolites are selectively required by NSCLC cells in response to RT exposure.

As SHMT2 is the main cancer-related enzyme responsible for ser/gly conversion and downstream synthesis of the pyrimidine nucleotide TMP, we measured SHMT2 protein expression by flow cytometry in response to irradiation across a NSCLC cell panel (*n* = 6). We observed a dose-dependent increase in SHMT2 protein expression after exposure to 4 Gy and 6 Gy for 24 h (*p* = 0.0113, *p* < 0.0001, respectively) (Fig. 2b, c). The increase of SHMT2 protein expression 24 h post-irradiation with 6 Gy was also confirmed by immunoblot in Calu-6, HCC15, NCI-H520, NCI-H125 and NCI-H1975 cell lines (Fig. 2d). NCI-H1299 cells did not show increased protein expression, however, this cell line displayed a baseline high metabolic flux of glucose towards ser/gly synthesis, which was further enhanced after irradiation (Supplementary Fig. S4C). In addition, the requirement for ser/gly pathway metabolites in response to RT was further validated using the isogenic model A549 with NKX2-1 overexpression. Our group has recently described that NKX2-1 induces dependency on de novo ser/gly synthesis to control ATP supply, nucleotide metabolism and redox homeostasis. A549 cells with overexpression of NKX2-1 exhibited decreased sensitivity to irradiation with higher proliferation rate after irradiation compared to control cells (Fig. 2e) [23]. These data indicate that RT results in an elevated dependency on SHMT2 activity and consequently, ser/gly synthesis and downstream metabolic products, with the usage of these metabolites to sustain NSCLC proliferation and RT recovery.

### Targeting intrinsic dependency on ser/gly synthesis synergizes with RT, inhibiting cell proliferation and clonogenicity of NSCLC cell lines

**Fig. 3:**
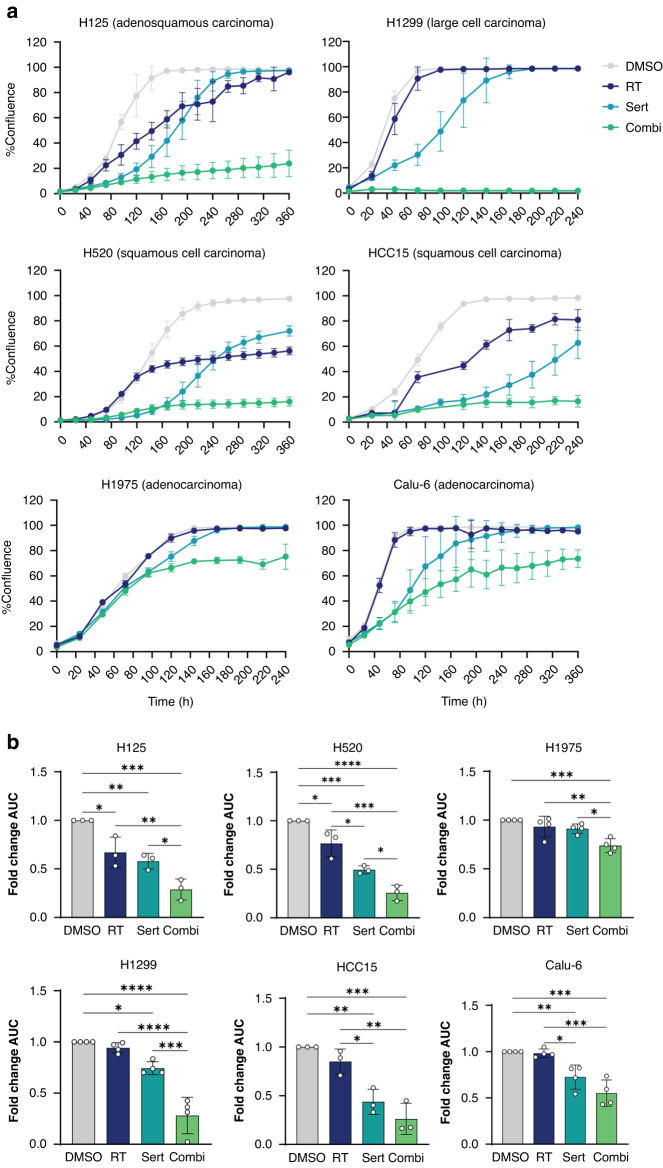
Targeting intrinsic dependency on ser/gly synthesis in combination with RT has synergistic effects in cell growth in NSCLC cell lines. **a** Growth curves of NSCLC cell lines treated with 10 µM sertraline and in combination with irradiation, 6 Gy. Calu-6 cell line was treated with 5 µM sertraline and 6 Gy irradiation. Data are presented as mean ± SD of 6 replicate wells and are representative of *n* ≥ 3 independent experiments. **b** Fold change of area under the curve (AUC) of cell growth curves of *n* ≥ 3 independent experiments. Data are represented as mean ± standard deviation. Individual dots represent independent observations. One-way ANOVA with Tukey’s multiple comparison test was used. Statistical analysis *p-value < 0.05, **p-value < 0.01, ***p-value < 0.001, ****p-value < 0.0001.

Since NSCLC cells seem to be dependent on ser/gly metabolism to recover and survive from RT induced cellular damage, we aimed to investigate whether these NSCLC cells are more sensitive to the combination of RT and ser/gly metabolism-targeting therapy. For this reason, we explored the efficacy of combining RT with sertraline, a repurposed SHMT inhibitor [19]. Incucyte confluence monitoring revealed that sertraline in combination with RT severely impaired the proliferation of NSCLC cell lines. We observed a complete inhibition of the cell proliferation of NCI-H1299, NCI-H125, NCI-H520, NCI-HCC15 with the combination treatment, which was slightly less robust in NCI-H1975, and Calu-6 cell lines. Interestingly, although sertraline as monotherapy caused a delay in NSCLC proliferation rate, these cells could recover over time (Fig. 3a). The analysis of the data via areas under the curve (AUC) confirmed a significantly stronger inhibition of proliferation by the combination of sertraline and RT as compared to sertraline as monotherapy in NCI-H1299, NCI-H125, NCI-H520 and NCI-H1975 (*p* = 0.0001*, p* = 0.0356*, p* = 0.0310*, p* = 0.0183, respectively) and as compared to RT monotherapy in NCI-H1299, NCI-H125, NCI-H520, HCC15, NCI-H1975 and Calu-6 (*p* < 0.0001*, p* = 0.0085*, p* = 0.0003*, p* = 0.0016, *p* = 0.0.0088 and *p* = 0.0003, respectively) (Fig. 3b). To address the degree of synergy between sertraline and irradiation, we determined the combination index (CI) using normalized values of area under the curve (AUC). We could observe a synergistic effect of the combination treatment in NCI-H1975 (CI = 0.58), Calu-6 (CI = 0.64) and NCI-H1299 (CI = 0.42); a moderate synergistic effect in NCI-H520 (CI = 0.83) and HCC15 (CI = 0.85); and a slight synergistic effect in NCI-H125 (CI = 0.86).

**Fig. 4:**
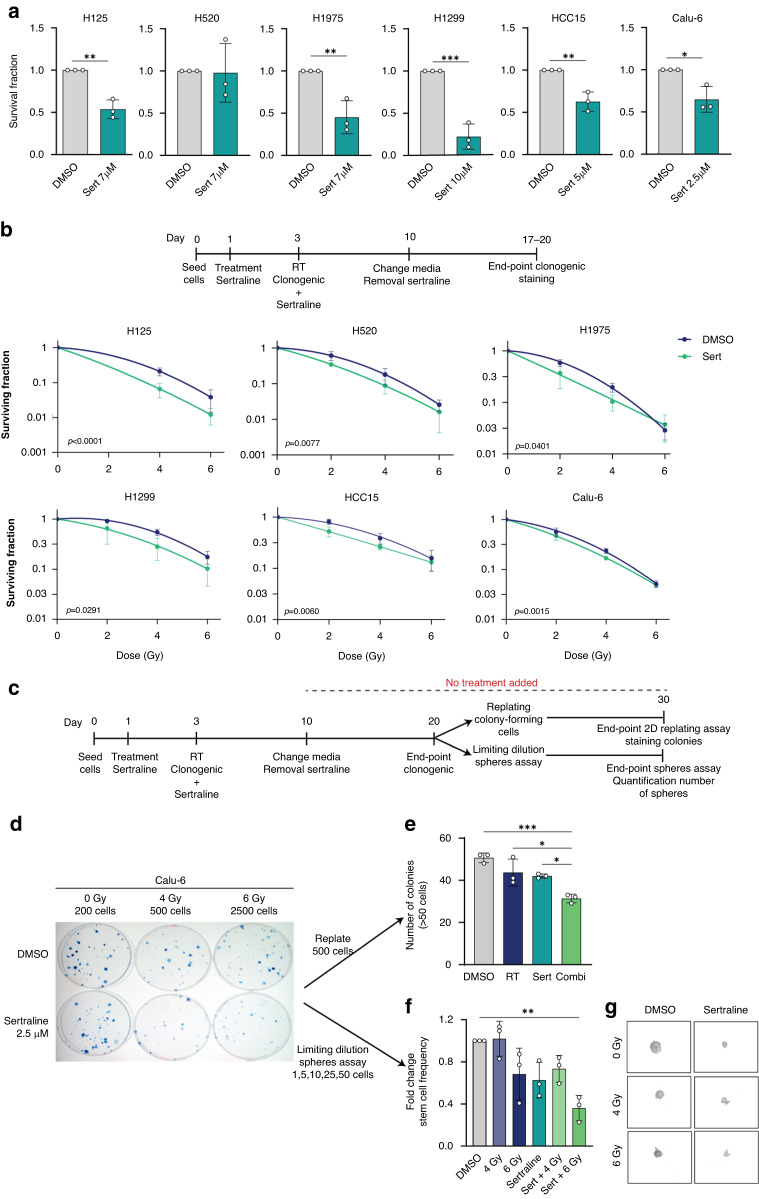
Targeting intrinsic dependency on ser/gly synthesis radio-sensitizes NSCLC cell lines and decreases their stemness. **a** Quantification of clonogenic assays as survival fraction of sertraline treatment relative to DMSO controls in NSCLC cell lines. After pre-treatment with sertraline for 48 h, single cells were seeded in DMSO, or sertraline containing media and left for 7 days. Media was changed after 7 days allowing the cells to grow without treatment for 7–10 more days; colonies were counted, calculating the survival fraction. Data are represented as mean ± standard deviation. Individual dots represent independent observations. P-values were calculated using an unpaired 2-tailed t-test. **b** Survival curves for NSCLC cells upon increasing doses of irradiation 2, 4 and 6 Gy, with and without sertraline. NSCLC cell lines were pre-treated with sertraline for 48 h and, subsequently exposed to 2, 4 and 6 Gy irradiation doses. Cells were dissociated and single cells seeded in DMSO, or sertraline containing media and left for 7 days. Media was changed after 7 days allowing the cells to grow without treatment for 7–10 more days. Survival fraction was determined relative to the non-irradiated cells with and without sertraline. Average results of *n* ≥ 3 experiments are shown, and data are fitted to a quadratic linear model. **c** Timeline showing the experimental procedure. A clonogenic as described above was performed, followed by a clonogenic replating or a limiting dilution sphere formation assay in the absence of any treatment. **d** Representative images of Calu-6 clonogenic assay. **e** Graph showing the number of colonies (>50 cells) after replating 500 cells from end-stage clonogenic surviving colonies of the Calu-6 cell line. One-way ANOVA with Tukey’s multiple comparison test was used. Data are represented as mean ± standard deviation. Individual dots represent different technical replicates. **f** Graph showing the fold change in stem cell frequency of the different originating groups compared to the control cells. One-way ANOVA with Dunnett’s multiple comparisons test was used. Data are represented as mean ± standard deviation. Individual dots represent *n* = 3 independent calculations from limiting dilution spheroid experiments. **g** Representative pictures at 20× magnification of Calu-6 spheres at day 30 after an initial seeding density of 25 cells/well and condition. Statistical analysis *p-value < 0.05, **p-value < 0.01, ***p-value < 0.001.

Furthermore, we explored the therapeutic efficacy in clonogenic assays using a linear quadratic model to monitor the surviving fraction, plotted in a logarithmic scale. We replaced the media after 7 days, allowing cells to grow and form colonies for another 7–10 days. We observed a significantly reduced clonogenic survival in NCI-H125, NCI-H1299, NCI-H1975, HCC15 and Calu-6 cell lines using sertraline as monotherapy (Fig. 4a). The combination of sertraline with RT showed an improved response compared to only irradiation in all NSCLC cell models, with a higher effect at low irradiation doses (Fig. 4b). Yet, the colonies in combination treatment at higher irradiation doses presented more dispersed colonies. Therefore, we performed a re-plating assay and a limiting dilution sphere formation assay for the cell line Calu-6, allowing us to investigate long-term intrinsic replating capacity and self-renewal of these end-stage clonogenic surviving cells (Fig. 4c). This analysis revealed a 40% reduction in the replating capacity of end-stage clonogenic surviving cells that were treated 20 days earlier with sertraline in combination with 4 Gy RT compared to DMSO control (DMSO vs Sert 4 Gy, Sert vs Sert 4 Gy, 4 Gy vs Sert 4 Gy, *p* = 0.0008, *p* = 0.0278, and *p* = 0.0129, respectively) (Fig. 4d, e). The limiting dilution sphere formation assay showed that the cells that were treated 20 days earlier with the combination of sertraline and 6 Gy irradiation were intrinsically rewired and presented a decreased sphere formation efficiency, indicating a 3-fold reduction and loss of long-term self-renewal capacity (*p* = 0.0061) (Fig. 4f). Overall, these in vitro data support that sertraline addition to RT is successful in both targeting the bulk cancer cell proliferation as well as targeting their cancer stem cell properties i.e., clonogenic and self-renewal capacity.

### The combination of sertraline and RT in NSCLC cell lines increases ROS levels

To further assess the impact of SHMT inhibition on NSCLC cellular responses to radiation, we analyzed cell cycle, DNA damage, cell death responses, and ROS levels. Firstly, we measured the cell cycle phases using propidium iodide staining of the DNA by flow cytometry. We observed an increased percentage of cells in G2/M phase 24 h post-RT in NCI-H125, NCI-H520, Calu-6 and NCI-HCC15 cell lines and a decreased percentage of cells in S phase in NCI-H125 and NCI-H520 cell lines (Supplementary Fig. S5A). In addition, the expression of the cell cycle inhibitor p21^CIP/WAF1^ was enhanced in NCI-H1299, NCI-H125, NCI-H520, Calu-6 and NCI-H1975 cell lines upon irradiation (Supplementary Fig. S5B) [33, 34]. Sertraline as monotherapy caused a significant reduction in the percentage of cells in S phase in the cell line NCI-H125 (*p* = 0.0020), which was further confirmed by BrdU incorporation in immunofluorescence stainings (*p* = 0.0048) (Supplementary Fig. S5C). Yet, different effects were observed in response to combination therapy across the NSCLC cell models. In NCI-H1299 and Calu-6, we observed a significant reduction of cells in S phase (*p* = 0.0011, *p* = 0.0153, respectively). In NCI-H1975 the combination treatment caused a significant arrest of cells in G2/M phase compared to irradiation as monotherapy (*p* = 0.0004). In contrast, NCI-H125, NCI-H520 and NCI-HCC15 showed a significant reduction of cells in G2/M phase compared to RT alone, which could indicate an abrogation of the G2/M cell cycle arrest needed to repair the radiation damage (Supplementary Fig. S5A) [35, 36]. Consistently, cell lines with abrogation of G2/M phase upon combination treatment, i.e., NCI-H520 and NCI-HCC15 also presented a reduction of the expression of p21^CIP/WAF1^ and p-ATM, the latter involved in the DNA damage response pathway (Supplementary Figs. S5B and S6A). Next, we investigated whether the combination treatment was inducing differences in residual DNA damage by analyzing γH2AX by flow cytometry. As expected, most of the irradiated cells, except NCI-H1975 and Calu-6, showed an increase in residual DNA damage 24 h post-RT, which was not further enhanced by the combination treatment (Supplementary Fig. S6B). Thereafter, we observed increased expression of cell death marker cleaved-caspase 3 upon sertraline as monotherapy in NCI-H125, NCI-H1975, NCI-H1299 and HCC15 cell lines, and the combination therapy in NCI-H125, NCI-H520, NCI-H1975, NCI-H1299 and HCC15 cell lines, highlighting the partial cytotoxic effect of sertraline and combination therapy (Supplementary Fig. S7). In summary, despite different responses in the individual NSCLC models, the general effect of sertraline in combination with irradiation that was observed in cell inhibitory effects and clonogenic assays might not be explained by effects on DNA damage.

**Fig. 5:**
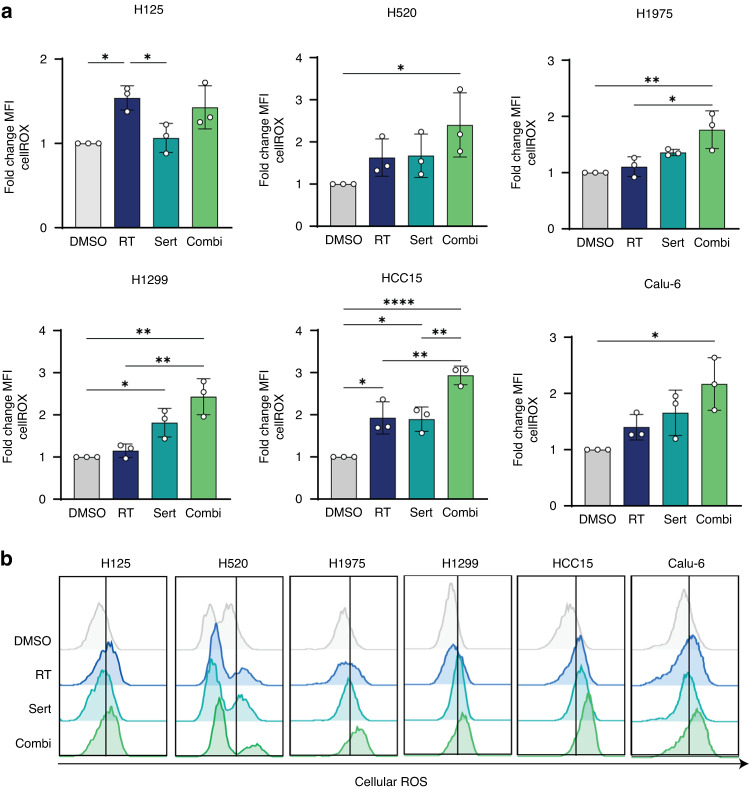
Targeting intrinsic dependency on ser/gly synthesis in combination with RT increases ROS levels in NSCLC cell lines. **a** NSCLC cell lines were pre-treated with sertraline for 48 h, and then irradiated with 6 Gy with and without sertraline. Cellular ROS levels were analyzed 24 h post-RT by flow cytometry using cellROX probe. The graphs represent the fold change of Mean Fluorescence Intensity (MFI) for *n* = 3 independent experiments. One-way ANOVA with Tukey’s multiple comparison test was used. Data are represented as mean ± standard deviation. Individual dots represent independent observations. Statistical analysis *p-value  <  0.05, **p-value  <  0.01, ****p-value < 0.0001. **b** Representative flow cytometry graphs of cellROX fluorescence intensity in NSCLC and cell lines in response to sertraline, irradiation, and the combination treatment.

Our metabolic analysis of NSCLC plasma samples suggested an important role of GSH during and after RT in patients with NSCLC. Radiation not only generates ROS, causing DNA damage and thereby cell death, but also increases endogenous ROS production [37–40]. For this reason, we explored ROS levels in NSCLC cell lines after irradiation and sertraline and combination treatment. We observed a 2-3-fold increase in cellular ROS in NCI-H520, NCI-H1975, NCI-H1299, HCC15 and Calu-6 upon the combination treatment (Fig. 5a, b). Even though treatment with sertraline as monotherapy caused an increase in ROS levels compared to control in NCI-H1299 and NCI-HCC15, these cell lines showed the highest ROS levels with the combination treatment, with significant differences between sertraline and the combination treatment in NCI-HCC15 (*p* = 0.0056) (Fig. 5a, b). In summary, the improved response to irradiation that we observed with the combination of sertraline and RT might be due to enhanced increase in ROS levels and oxidative stress in NSCLC cells, thereby eradicating their continuous proliferation and reducing their self-renewal ability.

### Targeting intrinsic dependency on ser/gly synthesis in combination with RT reduces tumor growth in vivo and affects the serum levels of glycine, threonine, and homocysteine

**Fig. 6:**
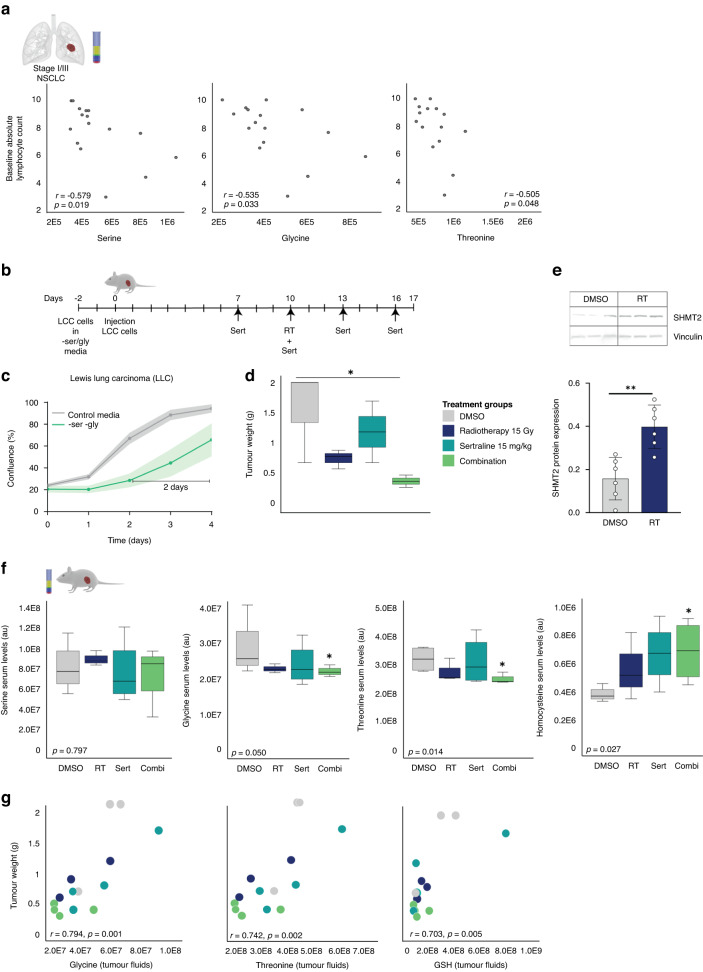
The combination treatment affects the serum levels of glycine and the downstream metabolite homocysteine and reduces LLC tumor growth in vivo. **a** Inverse correlations between baseline absolute lymphocyte counts before RT in patients with stage I/III NSCLC and ser/gly pathway metabolites in the blood plasma. Pearson’s correlations between metabolites (arbitrary units) and baseline absolute lymphocyte counts (10^3^/µl), *n* = 16. **b** Schematic representation of in vivo experiment and treatment schedule. LLC cells were cultured for 2 days in media depleted of serine and glycine to induce ser/gly synthesis. C57BL/6 mice were injected in the right flank with LLC cells. Half of the tumor bearing mice were locally irradiated with 15 Gy. All groups received intraperitoneal treatment injections, either DMSO (10%) or sertraline (15 mg/kg). Sertraline treatment was initiated 3 days prior to RT, i.e., on day 7, and repeated every 3 days. End-point tumors on day 17 were processed and used for further analyses. **c** Growth curves of LLC cells cultured in control media and media depleted of serine and glycine amino acids. Data are presented as mean ± SD of 6 replicate wells. **d** Box plot showing tumor growth as tumor weight in the different treatment groups: DMSO, RT 15 Gy, sertraline 15 mg/kg, and combination. Kruskal–Wallis test with a Dunn’s multiple comparisons test was performed. **e** SHMT2 protein expression on day 17 measured by immunoblot in *n* = 3 LLC tumors and n = 2 technical replicates. An unpaired two-tailed t test has been used to compare DMSO treated mice and irradiated tumors. **f** Box plots showing the serum levels of serine, glycine, threonine, and homocysteine in mice treated with DMSO, RT, sertraline as monotherapies and the combination of sertraline and RT. A Jonckheere-Terpstra test was performed. **g** Pearson correlations of glycine, threonine and GSH with tumor weight. Statistical analysis *p-value  <  0.05.

In our cohort of patients with NSCLC we observed negative associations between baseline serine, glycine and threonine plasma levels and circulating lymphocytes (Fig. 6a), suggesting that tumor derived systemic ser/gly pathway metabolites suppress the lymphoid system in humans. Moreover, ser/gly producing lung cancer cells induced an immune suppressive phenotype in a co-culture system ex vivo with mouse bone marrow cells. We observed a superior reduction in the amount of viable immune cells, as well as reductions in the percentage of CD4+ T-cells in co-cultures with ser/gly producing NKX2-1 lung cancer cells (Supplementary Fig. S8). Therefore, we hypothesized that sertraline treatment in combination with RT would not only target the tumor by SHMT inhibition to enhance sensitivity of NSCLC tumors to radiation in vivo but may also release the brake on the immune system. For that reason, we used an immune suppressive mouse lung cancer model, i.e., LLC, in immunocompetent C57BL/6 mice. After two days under ser/gly deprived conditions, LLC cells undergo metabolic reprogramming towards de novo ser/gly synthesis, becoming independent to the uptake of extracellular serine and glycine from their environment and increasing their proliferation rate. In addition, LLC cells showed sensitivity to sertraline treatment (Fig. 6b, c, Supplementary Fig. S9). C57BL/6 mice were subcutaneously transplanted with LLC lung tumor cells and treated with DMSO or sertraline every 3 days starting after 1 week of tumor progression. On day 10, half of the tumor bearing mice were locally irradiated with a single dose of 15 Gy, of which 4 animals received sertraline treatment and 4 received DMSO. We determined tumor growth at the end-time point, on day 17, by absolute tumor weights. While monotherapy was ineffective in targeting tumor progression, we observe a significant reduction in tumor growth in the group receiving sertraline together with RT (*p* = 0.042) (Fig. 6d, Supplementary Fig. S10). Analysis of the tumors confirmed once more the enhanced expression of SHMT2 in irradiated tumors compared to non-irradiated tumors, verifying their need for ser/gly pathway metabolites in response to irradiation (Fig. 6e).

Since sertraline targets SHMT systemically, we were interested in the metabolite profiles in the serum of mice with lung tumors that were treated with RT and sertraline as monotherapies, as well as the combination treatment. In support of our previous findings, the ser/gly pathway metabolites in the blood slightly drop even 10 days after RT, showing their consumption requirement for these metabolites to recover from RT. We observed a significant decrease in glycine serum levels upon the combination treatment, while serine levels were unchanged (*p* = 0.050 and *p* = 0.797, respectively), indicating that downstream glycine blood levels reduce once that tumors become dependent on consumption as well as production, the latter blocked by adjuvant sertraline (Fig. 6f). Threonine, which can act downstream of glycine, was decreased in the blood serum of mice within the combination treatment group (*p* = 0.014) (Fig. 6f) [41]. Homocysteine requires glycine to enable the synthesis of GSH, and due to the lack of glycine, homocysteine accumulates in the serum of combination treated mice (Fig. 6f, *p* = 0.027). Interestingly, analyses of intra-tumoral metabolites revealed positive correlations between glycine, threonine and GSH and the tumor weights (Fig. 6g, R = 0.794, *p* = 0.001; R = 0.742, *p* = 0.02; R = 0.703, *p* = 0.005, respectively). Collectively, these results indicate that the combination treatment restricts the tumor from RT-induced requirements for ser/gly pathway metabolites.

### The combination treatment alleviates the tumor immunosuppressive phenotype

**Fig. 7:**
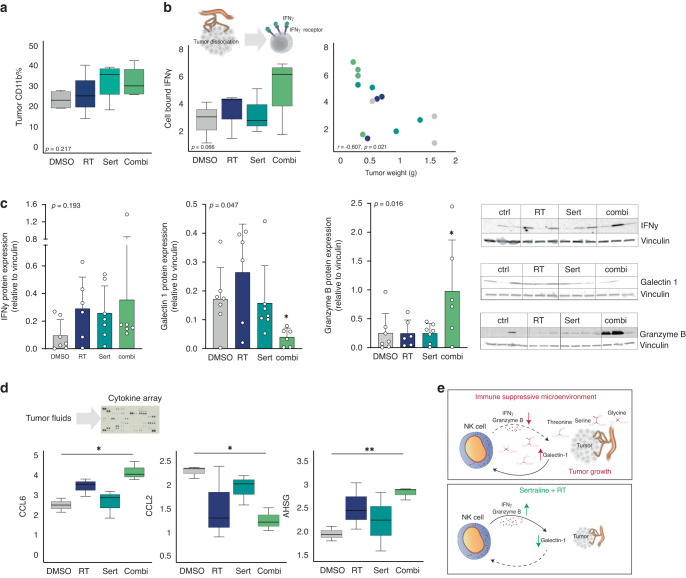
The combination treatment with sertraline and RT improves in vivo immune landscape. **a** Box plot showing the % of tumor CD11b measured by flow cytometry in the different treatment groups of dissociated mice tumors. **b** From left to right; box plot showing the % of cell bound IFN-γ measured by flow cytometry in dissociated tumors collected from mice treated with DMSO, RT, sertraline, and combination treatment. Pearson correlations of cell bound IFN-γ with tumor weight. **c** IFN-γ, galectin-1 and granzyme B protein expression measured by immunoblot in the tumors of *n* ≥ 3 mice for each treatment group and *n* = 2 technical replicates. A Jonckheere-Terpstra test was performed. **d** Cytokine analysis of tumor fluids from *n* ≥ 3 mice for each treatment group and *n* = 2 technical replicates. Welch’s ANOVA test with Dunnett’s T3 multiple comparisons test was used. **e** Schematic representation of how ser/gly metabolites influence NK cells. Decrease levels of galectin-1 enhances the tumor-killing effects of NK cells by releasing granzyme B. Statistical analysis *p-value < 0.05, **p-value < 0.01.

One of the challenges in cancer treatment is the immune suppression mediated by the tumor and its surrounding microenvironment, leading to poor therapeutic responsiveness. While the tetrameric form of SHMT2 participate in the one-carbon unit metabolism, the dimeric form of SHMT2 has been shown to enhance the IFN-γ immune response [42, 43]. In clinical setting, high IFN-γ expression is associated with improved outcomes in the response to immune checkpoint blockade [44, 45]. Therefore, we assessed the effects of sertraline in combination with RT on promoting an anti-tumor immune response. Tumors were mechanically dissociated, and the expression of immune markers on single tumor cells were analyzed by flow cytometry (Fig. 7a, b, Supplementary Fig. S11A). We observed that the % of CD11b+ cells within the tumor showed a slight increase with sertraline treatment (Fig. 7a). CD11b is a marker expressed on innate cell subtypes, such as macrophages, granulocytes, and natural killer (NK) cells, and previous studies have identified the activation of CD11b as a promising strategy to enhance adaptive anti-tumor immunity in lung cancer [46]. In addition, the levels of cell-bound IFN-γ showed a trend to be increased with the combination of sertraline and RT, which was inversely associated with tumor weight (R = −0.607 *p* = 0.021) (Fig. 7b). Immunoblot analysis confirmed the trend of IFN-γ levels to be enhanced in the tumors that were treated with sertraline and irradiation. Since NK cells represent the main effector cells in the innate immune response that generate IFN-γ, we further investigated NK-associated mechanisms [47, 48]. NK cells eliminate tumor cells by releasing cytotoxic granules containing granzyme B, and among the immunosuppressive mechanisms associated to NK cells, secreted galectin-1 hinders the tumor-killing effects of these cells by reducing the release of granzyme B [49, 50]. Interestingly, the analysis of the tumors by immunoblot revealed decreased levels of galectin-1 and a subsequent elevated levels of granzyme B in tumors that were treated with the combination of sertraline and irradiation (*p* = 0.047, *p* = 0.016, respectively) (Fig. 7c). These findings suggest that sertraline in combination with irradiation releases the NK-linked immunosuppressive phenotype in lung cancer due to the restriction of ser/gly pathway metabolite availability.

Next, we performed a cytokine array to gain more insight into the immune landscape in the tumor microenvironment following the combined administration of sertraline and RT (Supplementary Fig. S11B). The cytokine analysis showed a significant increase of chemokine (C-C motif) ligand 6 (CCL6) levels with the combination treatment (*p* = 0.0327) (Fig. 7d). Previous studies in mouse models demonstrated that CCL6 promotes innate immunity via immune cell activation, and serves as a chemoattractant for CD11b+ effector immune cells with the enhanced ability to generate IFN-γ, such as NK cells [51, 52]. On the other hand, the levels of the C-C motif chemokine ligand 2 (CCL2) were downregulated following sertraline and irradiation treatment (*p* = 0.0279). CCL2 attracts immunosuppressive cells to the tumor microenvironment, thereby favoring cancer development [53–55]. Anti-CCL2 therapy has been proposed to be a potential strategy to enhance NK cell activity, leading to more effective anti-tumor responses [56]. In addition, we observed elevated levels of alpha 2-HS glycoprotein (AHSG) upon combination treatment (*p* = 0.0076). While the functions of AHSG remain largely unknown, it is suspected to play an important role in blocking the transforming growth factor (TGF)-β, which suppresses the activation and functions of NK cells [57–59].

Taken together, these results show that the inhibition of SHMT with sertraline in combination with RT reshapes the tumor microenvironment and alleviates the immunosuppressive phenotype, showing an improved NK-associated anti-tumor immune signature and tumor growth delay. Therefore, this study emphasizes the high potential for additional sertraline treatment in patients with NSCLC that receive RT as standard-of-care.

## Discussion

The findings in this study show that pharmacological targeting of ser/gly synthesis using sertraline in NSCLC models can reduce cancer cell survival and stem cell self-renewal capabilities, with an enhanced radiation response. These changes are associated with improved NK cell signatures and cytokines in the tumor microenvironment that lead to enhanced anti-tumor immune responses.

Metabolic rewiring is a well-described hallmark of cancer cells, which supports cancer cell survival, proliferation, invasion and resistance to cancer treatments [60]. These tumor-associated metabolites are well reflected in the blood. Blood plasma/serum samples are easily accessible clinically and allow to investigate the metabolic changes in patients with cancer and in response to cancer treatments. One of the main aims is to identify biomarkers that can reflect cancer status in the early stages as well as predict prognosis and responses to cancer treatments. In this study, we investigated ser/gly metabolism in plasma samples of patients with stage I and III NSCLC in order to identify cancer-associated metabolic changes in response to RT. We identified a significant decrease in GSH in patients with stage I/III NSCLC during and after RT. Downstream of the ser/gly synthesis pathway, GSH is a reductive antioxidant that controls redox homeostasis and protects from chemo-radiation therapy induced oxidative toxicity. Similar to our observations, a decrease in GSH plasma levels has been previously observed in patients with breast cancer during doxorubicin chemotherapy treatment [61]. GSH is derived from homocysteine, and both were decreased in the patients with NSCLC analyzed in this study. In previous studies, serine and glycine, as well as pyrimidine and purine metabolism alterations have been identified in the serum metabolome of patients with prostate cancer before and after treatment with stereotactic body radiation therapy (SBRT), and combination of intensity-modulated radiation therapy (IMRT) and SBRT [62]. The patients with NSCLC included in this study showed an overall average nutritional status based on their body mass index before treatment. However, we cannot exclude that treatment related malnutrition in some patients might have affected the metabolite blood plasma levels during and after treatment. There are also some limitations in this study, such as the relatively small number of patients and lack of samples of patients with a more advanced stage of NSCLC, such as stage IV, since ser/gly metabolism hyperactivation is associated to poor prognostic advanced NSCLC.

The lower intracellular levels of serine and glycine in cancer cells that we observed in vitro indicate a high turnover with increased consumption and usage of these amino acids in downstream biosynthetic pathways, which is supported by the enhanced SHMT2 expression in response to RT. In addition, the most RT resistant NSCLC cell model showed elevated ser/gly synthesis in response to RT treatment. These mechanisms were not exploited by normal lung cells, highlighting the selective adaptation towards ser/gly metabolism in cancer cells. Consequently, we explored whether NSCLC cells were more sensitive to the combination of RT and ser/gly metabolism-targeted therapies. Since the more recently developed PHGDH and SHMT inhibitors are not yet clinically applicable in humans, sertraline reflects a perfect candidate for ser/gly dependent cancers by targeting SHMT [19, 63–65]. The dosage of sertraline used in our animal model is equivalent to the dosage received by patients for the indication of depression. Yet, we administer sertraline only once every 3 days to reach a peak dosage, while patients have a daily oral intake with a maximum of 250 mg. This means, 22 mg/kg per week for a person of 80 kg, while we administered 15 mg/kg twice a week. Considering that mice show ~7-fold higher metabolic turnover, this in vivo study represents a treatment model that is achievable for the treatment of patients with NSCLC in the clinic. Moreover, this study shows that glycine, GSH, threonine, and homocysteine could function as adequate biomarkers to monitor therapy effectiveness in the patients’ blood.

We revealed that sertraline acts synergistically in NSCLC cell lines in combination with RT, by inhibition of cell growth, as well as by reducing clonogenic survival, observing no differences associated to the genetic background of these NSCLC cell models. In addition, a colony replating assay and sphere formation assay showed a decreased ability to repopulate colonies and form spheres even long after the treatments have been removed, reducing their intrinsic stem cell capabilities. Interestingly, our in vitro data showed a significant increment of ROS after the combination treatment. Mechanisms of radio-resistance in cancer stem cells are associated with higher antioxidant levels that are capable to scavenge ROS, since low levels of ROS are associated with maintaining their stemness [66–69]. Of note, we already described that sertraline as monotherapy decreases GSH levels [19]. The inhibition of serine and glycine bidirectional conversion by sertraline prior irradiation reduces the pool of nucleotides, as we previously observed, and concurrent and post-treatment with sertraline might hinder the synthesis of the antioxidant GSH and NADPH production, which exposes cancer cells to the accumulation of endogenous ROS and radiation-induced ROS causing severe oxidative stress [70].

In vivo, we validated a significant reduction in tumor growth in the group receiving sertraline together with RT, which was accompanied with reduced glycine/threonine and an accumulation of homocysteine blood serum levels. These results indicate a blockage in the pathway of GSH synthesis and therefore could explain the build-up of oxidative stress that has been observed upon the combination treatment in vitro. On the other hand, we identified a trend of methionine sulfoxide levels to be decreased in the serum of the mice treated with the combination treatment, which could represent a compensatory mechanism in response to the accumulation of homocysteine (*p* = 0.051) (Supplementary Fig. S12). Homocysteine can be diverted into the trans-sulfuration pathway, being the precursor of cysteine, which will be used in GSH production together with glycine and glutamate. However, homocysteine can also be re-methylated to methionine in the methionine cycle. Methionine provides antioxidant defense through reversive oxidation and reduction. The interaction with ROS causes the oxidation of methionine into methionine sulfoxide. Methionine sulfoxide can be reduced back to methionine, creating an efficient system to scavenge ROS. This mechanism would represent another antioxidant system as an alternative to GSH production to protect against oxidative stress that should be taken into account in future studies [71].

A recent publication has identified that ser/gly starvation sensitizes colorectal, breast and pancreatic cancer models to RT, which further supports our observations for ser/gly uptake dependent cancers. They also showed the high impact of RT in metabolic pathways that are involved in antioxidant and DNA-damage repair, such as, GSH synthesis and turnover, and nucleotide metabolism [72].

Of note, in the patients with NSCLC, we observed that baseline serine, glycine and threonine plasma levels were negatively associated to patient’s lymphocyte counts. Previously, *SHMT2* expression has been shown to be inversely associated with tumor-infiltrating lymphocytes in the tumor microenvironment of patients with lung adenocarcinoma, using TIMER and TISIDB online databases [73, 74]. In addition, we have observed the induction of an immune suppressive phenotype by ser/gly producing lung cancer cells, using a co-culture system ex vivo. Interestingly, our results showed for the first time in-depth the impact of targeting ser/gly metabolism using sertraline in combination with RT on the immune tumor microenvironment. In vivo, the combination treatment could remodel the immune landscape, reducing levels of CCL2 cytokine, which has been linked to immune suppression and poor prognosis, and increasing the levels of cytokines that enhance anti-tumor responses, such as CCL6 [51, 75, 76]. These changes are associated with NK cells, as supported by the decreased levels of the immune suppressive protein galectin-1 and the accompanied elevated levels of granzyme B. Galectin-1 favors tumor growth by impairing the anti-tumor effects of NK cells. It has been shown that glioma cells overexpressing galectin-1 are capable to evade NK immune surveillance, whereas the lack of galectin-1 leads to accentuated tumor-killing effects of NK cells by releasing granzyme B [50, 77]. Hence, these observations suggest that tumor derived ser/gly metabolism could influence an immune suppressive phenotype, which could be alleviated by neo(adjuvant) and concurrent sertraline treatment. Notably, LLC has been proven to be a highly aggressive and poorly immunogenic tumor model, unresponsive to mono- and bi-modal therapies, such as radiotherapy and immune checkpoint blockade [78]. Therefore, the anti-tumor immune related findings of this study underscore the potential of combining sertraline and RT as therapeutic strategy for immunosuppressive tumors. Further investigations are necessary to gain more insight into ser/gly metabolism and its contribution to the tumor microenvironment, including the relation between ser/gly pathway derived- “oncometabolites” and immune suppression at single cell level, both spatial and temporal, local and systemically.

In summary, our findings highlight that targeting ser/gly metabolism using sertraline radio-sensitizes and improves the therapeutic outcome in NSCLC. The observation of SHMT2 upregulation in response to RT also applies to multiple myeloma models, underscoring a broader application of sertraline treatment across cancers (Supplementary Fig. S13). In addition, this study represents the starting point for future investigations targeting ser/gly metabolism in combination with RT to simultaneously enhance immunotherapy responses in NSCLC and may set the basis for future clinical trials.

### Supplementary information

Supplementary data file

## Data Availability

The datasets generated during and/or analyzed during this study are available from the corresponding author on reasonable request.

## References

[1] Herbst RS, Morgensztern D, Boshoff C (2018). The biology and management of non-small cell lung cancer. Nature.

[2] Hu Y, Smyth GK (2009). ELDA: extreme limiting dilution analysis for comparing depleted and enriched populations in stem cell and other assays. J Immunol Methods.

[3] Gray JE, Villegas A, Daniel D, Vicente D, Murakami S, Hui R (2020). Three-year overall survival with durvalumab after chemoradiotherapy in stage III NSCLC—update from PACIFIC. J Thorac Oncol.

[4] Hirsch FR, Scagliotti GV, Mulshine JL, Kwon R, Curran WJ, Wu Y-L (2017). Lung cancer: current therapies and new targeted treatments. Lancet.

[5] Sánchez-Castillo A, Vooijs M, Kampen KR (2021). Linking serine/glycine metabolism to radiotherapy resistance. Cancers.

[6] Kerr EM, Gaude E, Turrell FK, Frezza C, Martins CP (2016). Mutant Kras copy number defines metabolic reprogramming and therapeutic susceptibilities. Nature.

[7] Shackelford DB, Shaw RJ (2009). The LKB1–AMPK pathway: metabolism and growth control in tumour suppression. Nat Rev Cancer.

[8] Kottakis F, Nicolay BN, Roumane A, Karnik R, Gu H, Nagle JM (2016). LKB1 loss links serine metabolism to DNA methylation and tumorigenesis. Nature.

[9] Ferretta A, Maida I, Guida S, Azzariti A, Porcelli L, Tommasi S (2016). New insight into the role of metabolic reprogramming in melanoma cells harboring BRAF mutations. Biochim Biophys Acta Mol Cell Res.

[10] Sun WY, Kim HM, Jung W-H, Koo JS (2016). Expression of serine/glycine metabolism-related proteins is different according to the thyroid cancer subtype. J Transl Med.

[11] Ross KC, Andrews AJ, Marion CD, Yen TJ, Bhattacharjee V (2017). Identification of the serine biosynthesis pathway as a critical component of BRAF inhibitor resistance of melanoma, pancreatic, and non–small cell lung cancer cells. Mol Cancer Therapeutics.

[12] Zeng Y, Zhang J, Xu M, Chen F, Zi R, Yue J (2021). Roles of Mitochondrial Serine Hydroxymethyltransferase 2 (SHMT2) in Human Carcinogenesis. J Cancer.

[13] Woo CC, Chen WC, Teo XQ, Radda GK, Lee PTH (2016). Downregulating serine hydroxymethyltransferase 2 (SHMT2) suppresses tumorigenesis in human hepatocellular carcinoma. Oncotarget.

[14] Du J, Huang Y, Jing S, Pei Y, Qian Y, Zeng Y (2022). Serine hydroxymethyltransferase 2 predicts unfavorable outcomes in multiple cancer: a systematic review and meta-analysis. Transl Cancer Res.

[15] Geeraerts SL, Heylen E, De Keersmaecker K, Kampen KR (2021). The ins and outs of serine and glycine metabolism in cancer. Nat Metab.

[16] Zhang B, Zheng A, Hydbring P, Ambroise G, Ouchida AT, Goiny M (2017). PHGDH defines a metabolic subtype in lung adenocarcinomas with poor prognosis. Cell Rep.

[17] Liao L, Yu H, Ge M, Zhan Q, Huang R, Ji X (2019). Upregulation of phosphoserine phosphatase contributes to tumor progression and predicts poor prognosis in non‐small cell lung cancer patients. Thorac Cancer.

[18] Locasale JW (2013). Serine, glycine and one-carbon units: cancer metabolism in full circle. Nat Rev Cancer.

[19] Geeraerts SL, Kampen KR, Rinaldi G, Gupta P, Planque M, Louros N (2021). Repurposing the antidepressant sertraline as SHMT inhibitor to suppress serine/glycine synthesis–addicted breast tumor growth. Mol Cancer Therapeutics.

[20] De Vane CL, Liston HL, Markowitz JS (2002). Clinical pharmacokinetics of sertraline. Clin Pharmacokinetics.

[21] Mandrioli R, Mercolini L, Raggi MA (2013). Evaluation of the pharmacokinetics, safety and clinical efficacy of sertraline used to treat social anxiety. Expert Opin drug Metab Toxicol.

[22] Owens MJ, Morgan WN, Plott SJ, Nemeroff CB (1997). Neurotransmitter receptor and transporter binding profile of antidepressants and their metabolites. J Pharmacol Exp Therapeutics.

[23] Heylen E, Verstraete P, Van Aerschot L, Geeraerts SL, Venken T, Timcheva K, et al. Transcription factor NKX2–1 drives serine and glycine synthesis addiction in cancer. Brit J Cancer. 2023:128:1862–78. 10.1038/s41416-023-02216-y PMC10147615 36932191

[24] Kampen KR, Fancello L, Girardi T, Rinaldi G, Planque M, Sulima SO (2019). Translatome analysis reveals altered serine and glycine metabolism in T-cell acute lymphoblastic leukemia cells. Nat Commun.

[25] Elia I, Haigis MC (2021). Metabolites and the tumour microenvironment: from cellular mechanisms to systemic metabolism. Nat Metab.

[26] Vaes RD, Reynders K, Sprooten J, Nevola KT, Rouschop KM, Vooijs M (2021). Identification of potential prognostic and predictive immunological biomarkers in patients with stage I and stage III Non-Small Cell Lung Cancer (NSCLC): a prospective exploratory study. Cancers.

[27] Foucquier J, Guedj M (2015). Analysis of drug combinations: current methodological landscape. Pharmacol Res Perspect.

[28] Dubois L, Biemans R, Reniers B, Bosmans G, Trani D, Podesta M (2015). High dose rate and flattening filter free irradiation can be safely implemented in clinical practice. Int J Radiat Biol.

[29] Montrose DC, Saha S, Foronda M, McNally EM, Chen J, Zhou XK (2021). Exogenous and endogenous sources of serine contribute to colon cancer metabolism, growth, and resistance to 5-fluorouracil. Cancer Res.

[30] Blount BC, Mack MM, Wehr CM, MacGregor JT, Hiatt RA, Wang G (1997). Folate deficiency causes uracil misincorporation into human DNA and chromosome breakage: implications for cancer and neuronal damage. Proc Natl Acad Sci.

[31] Chen C-W, Tsao N, Huang L-Y, Yen Y, Liu X, Lehman C (2016). The impact of dUTPase on ribonucleotide reductase-induced genome instability in cancer cells. Cell Rep.

[32] Brown KK, Spinelli JB, Asara JM, Toker A (2017). Adaptive reprogramming of de novo pyrimidine synthesis is a metabolic vulnerability in triple-negative breast cancer. Cancer Discov.

[33] Niculescu AB, Chen X, Smeets M, Hengst L, Prives C, Reed SI (1998). Effects of p21Cip1/Waf1 at both the G1/S and the G2/M cell cycle transitions: pRb is a critical determinant in blocking DNA replication and in preventing endoreduplication. Mol Cell Biol.

[34] Georgakilas AG, Martin OA, Bonner WM (2017). p21: a two-faced genome guardian. Trends Mol Med.

[35] Anastasov N, Höfig I, Vasconcellos IG, Rappl K, Braselmann H, Ludyga N (2012). Radiation resistance due to high expression of miR-21 and G2/M checkpoint arrest in breast cancer cells. Radiat Oncol.

[36] Playle L, Hicks D, Qualtrough D, Paraskeva C (2002). Abrogation of the radiation-induced G2 checkpoint by the staurosporine derivative UCN-01 is associated with radiosensitisation in a subset of colorectal tumour cell lines. Br J cancer.

[37] Azzam EI, Jay-Gerin J-P, Pain D (2012). Ionizing radiation-induced metabolic oxidative stress and prolonged cell injury. Cancer Lett.

[38] Tulard A, Hoffschir F, de Boisferon FH, Luccioni C, Bravard A (2003). Persistent oxidative stress after ionizing radiation is involved in inherited radiosensitivity. Free Radic Biol Med.

[39] Kim EM, Yang HS, Kang SW, Ho J-N, Lee SB, Um H-D (2008). Amplification of the γ-irradiation-induced cell death pathway by reactive oxygen species in human U937 cells. Cell Signal.

[40] Riley P (1994). Free radicals in biology: oxidative stress and the effects of ionizing radiation. Int J Radiat Biol.

[41] Shyh-Chang N, Locasale JW, Lyssiotis CA, Zheng Y, Teo RY, Ratanasirintrawoot S (2013). Influence of threonine metabolism on S-adenosylmethionine and histone methylation. Science.

[42] Wu Q, Chen X, Li J, Sun S (2020). Serine and metabolism regulation: a novel mechanism in antitumor immunity and senescence. Aging Dis.

[43] Walden M, Tian L, Ross RL, Sykora UM, Byrne DP, Hesketh EL (2019). Metabolic control of BRISC–SHMT2 assembly regulates immune signalling. Nature.

[44] Higgs BW, Morehouse CA, Streicher K, Brohawn PZ, Pilataxi F, Gupta A (2018). Interferon gamma messenger RNA Signature in tumor biopsies predicts outcomes in patients with non–small cell lung carcinoma or urothelial cancer treated with durvalumab. Clin Cancer Res.

[45] Karachaliou N, Gonzalez-Cao M, Crespo G, Drozdowskyj A, Aldeguer E, Gimenez-Capitan A, et al. Interferon gamma, an important marker of response to immune checkpoint blockade in non-small cell lung cancer and melanoma patients. Therapeutic Adv Med Oncol. 2018;10:1758834017749748. 10.1177/1758834017749748 PMC5784541 29383037

[46] Geraghty T, Rajagopalan A, Aslam R, Pohlman A, Venkatesh I, Zloza A (2020). Positive allosteric modulation of CD11b as a novel therapeutic strategy against lung cancer. Front Oncol.

[47] Vivier E, Raulet DH, Moretta A, Caligiuri MA, Zitvogel L, Lanier LL (2011). Innate or adaptive immunity? The example of natural killer cells. Science.

[48] Vivier E, Tomasello E, Baratin M, Walzer T, Ugolini S (2008). Functions of natural killer cells. Nat Immunol.

[49] Yu X, Qian J, Ding L, Yin S, Zhou L, Zheng S (2023). Galectin-1: a traditionally immunosuppressive protein displays context-dependent capacities. Int J Mol Sci.

[50] Shah D, Comba A, Faisal SM, Kadiyala P, Baker GJ, Alghamri MS (2021). A novel miR1983-TLR7-IFNβ circuit licenses NK cells to kill glioma cells, and is under the control of galectin-1. Oncoimmunology.

[51] Nardi V, Naveiras O, Azam M, Daley GQ (2009). ICSBP-mediated immune protection against BCR-ABL–induced leukemia requires the CCL6 and CCL9 chemokines. Blood..

[52] Coelho AL, Schaller MA, Benjamim CF, Orlofsky AZ, Hogaboam CM, Kunkel SL (2007). The chemokine CCL6 promotes innate immunity via immune cell activation and recruitment. J Immunol.

[53] Zalfa C, Paust S (2021). Natural killer cell interactions with myeloid derived suppressor cells in the tumor microenvironment and implications for cancer immunotherapy. Front Immunol.

[54] Wang Y, Zhang X, Yang L, Xue J, Hu G (2018). Blockade of CCL2 enhances immunotherapeutic effect of anti-PD1 in lung cancer. J bone Oncol.

[55] Chang AL, Miska J, Wainwright DA, Dey M, Rivetta CV, Yu D (2016). CCL2 produced by the glioma microenvironment is essential for the recruitment of regulatory T cells and myeloid-derived suppressor cells. Cancer Res.

[56] Sceneay J, Chow MT, Chen A, Halse HM, Wong CS, Andrews DM (2012). Primary tumor hypoxia recruits CD11b+/Ly6Cmed/Ly6G+ immune suppressor cells and compromises NK cell cytotoxicity in the premetastatic niche. Cancer Res.

[57] Swallow CJ, Partridge EA, Macmillan JC, Tajirian T, DiGuglielmo GM, Hay K (2004). α2HS-glycoprotein, an antagonist of transforming growth factor β in vivo, inhibits intestinal tumor progression. Cancer Res.

[58] Viel S, Marçais A, Guimaraes FS-F, Loftus R, Rabilloud J, Grau M (2016). TGF-β inhibits the activation and functions of NK cells by repressing the mTOR pathway. Sci Signal.

[59] Slattery K, Woods E, Zaiatz-Bittencourt V, Marks S, Chew S, Conroy M, et al. TGFβ drives NK cell metabolic dysfunction in human metastatic breast cancer. J Immuno Therapy Cancer. 2021;9:e002044. 10.1136/jitc-2020-002044 PMC7878131 33568351

[60] Hanahan D (2022). Hallmarks of cancer: new dimensions. Cancer Discov.

[61] Panis C, Herrera A, Victorino V, Campos F, Freitas L, De Rossi T (2012). Oxidative stress and hematological profiles of advanced breast cancer patients subjected to paclitaxel or doxorubicin chemotherapy. Breast Cancer Res Treat.

[62] Nalbantoglu S, Abu-Asab M, Suy S, Collins S, Amri H (2019). Metabolomics-based biosignatures of prostate cancer in patients following radiotherapy. Omics A J Integr Biol.

[63] Witschel MC, Rottmann M, Schwab A, Leartsakulpanich U, Chitnumsub P, Seet M (2015). Inhibitors of plasmodial serine hydroxymethyltransferase (SHMT): cocrystal structures of pyrazolopyrans with potent blood-and liver-stage activities. J Med Chem.

[64] Mullarky E, Lucki NC, Zavareh RB, Anglin JL, Gomes AP, Nicolay BN (2016). Identification of a small molecule inhibitor of 3-phosphoglycerate dehydrogenase to target serine biosynthesis in cancers. Proc Natl Acad Sci.

[65] Schwertz G, Witschel MC, Rottmann M, Bonnert R, Leartsakulpanich U, Chitnumsub P (2017). Antimalarial inhibitors targeting serine hydroxymethyltransferase (SHMT) with in vivo efficacy and analysis of their binding mode based on X-ray cocrystal structures. J Med Chem.

[66] Diehn M, Cho RW, Lobo NA, Kalisky T, Dorie MJ, Kulp AN (2009). Association of reactive oxygen species levels and radioresistance in cancer stem cells. Nature.

[67] Ding S, Li C, Cheng N, Cui X, Xu X, Zhou G. Redox regulation in cancer stem cells. Oxid Med Cell Longev. 2015;2015:750798. 10.1155/2015/750798 PMC4529979 26273424

[68] Ryoo I-g, Lee S-h, Kwak M-K. Redox modulating NRF2: a potential mediator of cancer stem cell resistance. Oxid Med Cell Longev. 2016;2016:2428153. 10.1155/2016/2428153 PMC4670665 26682001

[69] Chang C-W, Chen Y-S, Chou S-H, Han C-L, Chen Y-J, Yang C-C (2014). Distinct subpopulations of head and neck cancer cells with different levels of intracellular reactive oxygen species exhibit diverse stemness, proliferation, and chemosensitivity. Cancer Res.

[70] Yoshida T, Goto S, Kawakatsu M, Urata Y, Li T-s (2012). Mitochondrial dysfunction, a probable cause of persistent oxidative stress after exposure to ionizing radiation. Free Radic Res.

[71] Cabreiro F, Picot CR, Perichon M, Castel J, Friguet B, Petropoulos I (2008). Overexpression of mitochondrial methionine sulfoxide reductase B2 protects leukemia cells from oxidative stress-induced cell death and protein damage. J Biol Chem.

[72] Falcone M, Uribe AH, Papalazarou V, Newman AC, Athineos D, Stevenson K, et al. Sensitisation of cancer cells to radiotherapy by serine and glycine starvation. Brit J Cancer. 2022;127:1773–86. 10.1038/s41416-022-01965-6 PMC9643498 36115879

[73] Usman M, Hameed Y, Ahmad M, Iqbal MJ, Maryam A, Mazhar A, et al. SHMT2 is associated with tumor purity, CD8+ T immune cells infiltration, and a novel therapeutic target in four different human cancers. Curr Mol Med. 2023:23;161–76. 10.2174/1566524022666220112142409 35023455

[74] Luo L, Zheng Y, Lin Z, Li X, Li X, Li M, et al. Identification of SHMT2 as a potential prognostic biomarker and correlating with immune infiltrates in lung adenocarcinoma. J Immunol Res. 2021;2021:6647122. 10.1155/2021/6647122 PMC8049788 33928169

[75] Karan D. CCL23 in balancing the act of endoplasmic reticulum stress and antitumor immunity in hepatocellular carcinoma. Front Oncol. 2021;11:727583. 10.3389/fonc.2021.727583 PMC8522494 34671553

[76] Li L, Liu YD, Zhan YT, Zhu YH, Li Y, Xie D (2018). High levels of CCL2 or CCL4 in the tumor microenvironment predict unfavorable survival in lung adenocarcinoma. Thorac Cancer.

[77] Baker GJ, Chockley P, Yadav VN, Doherty R, Ritt M, Sivaramakrishnan S (2014). Natural killer cells eradicate galectin-1–deficient glioma in the absence of adaptive immunity. Cancer Res.

[78] Pimentel VO, Marcus D, van der Wiel AM, Lieuwes NG, Biemans R, Lieverse RI, et al. Releasing the brakes of tumor immunity with anti-PD-L1 and pushing its accelerator with L19–IL2 cures poorly immunogenic tumors when combined with radiotherapy. J. Immunotherapy Cancer. 2021;9:e001764. 10.1136/jitc-2020-001764 PMC7944996 33688020

