# Correction: Targeting serine/glycine metabolism improves radiotherapy response in non-small cell lung cancer

**DOI:** 10.1038/s41416-024-02603-z

**Published:** 2024-02-12

**Authors:** Anaís Sánchez-Castillo, Elien Heylen, Judith Hounjet, Kim G. Savelkouls, Natasja G. Lieuwes, Rianne Biemans, Ludwig J. Dubois, Kobe Reynders, Kasper M. Rouschop, Rianne D. W. Vaes, Kim De Keersmaecker, Maarten Lambrecht, Lizza E. L. Hendriks, Dirk K. M. De Ruysscher, Marc Vooijs, Kim R. Kampen

**Affiliations:** 1 Department of Radiation Oncology (MAASTRO), GROW School for Oncology and Reproduction, Maastricht University Medical Center+, Maastricht, The Netherlands; 2 Department of Oncology, Laboratory for Disease Mechanisms in Cancer, KU Leuven, and Leuven Cancer Institute (LKI), Herestraat 49, 3000 Leuven, Belgium; 3 Department of Precision Medicine, The M-Lab, GROW School for Oncology and Reproduction, Maastricht University, Maastricht, The Netherlands; 4 Department of Oncology, Experimental Radiation Oncology, KU Leuven, and Leuven Cancer Institute (LKI), Herestraat 49, 3000 Leuven, Belgium; 5 Department of Radiation Oncology, University Hospital Leuven, Leuven, Belgium; 6 Department of Pulmonology, GROW School for Oncology and Reproduction, Maastricht University Medical Center+, Maastricht, The Netherlands

**Keywords:** Cancer therapy, Lung cancer, Cancer metabolism

Correction to: *British Journal of Cancer* 10.1038/s41416-023-02553-y, published online 30 December 2023

In this article the funding section has been omitted during the production process. It should read:

Funding information

ASC received funding from the European Union’s Horizon 2020 research and innovation programme under the Marie Skłodowska-Curie grant agreement No # 860245 (to MV). E.H. was supported by an aspirant FWO fellowship 1106119N. KRK received funding to perform metabolomics mass spectrometry analysis, which was funded by a research grant from FWO (FWO KAN2018 871 1501419N), further experiments were supported by the KNAW early career award 2022 and FEBS excellence award 2022 (to KRK).

The original article has been corrected.

