# Supplementary material for: Targeting serine/glycine metabolism improves radiotherapy response in non-small cell lung cancer
Source: Br J Cancer. 2023 Dec 30;130(4):568–84. doi: 10.1038/s41416-023-02553-y (PMC10876524; doi:10.1038/s41416-023-02553-y)
Supplement: Supplementary file 1 — Supplementary data file [file 41416_2023_2553_MOESM1_ESM.docx]

Targeting serine/glycine metabolism improves radiotherapy response in Non-Small Cell Lung Cancer

**Running title: SHMT inhibition improves radiotherapy efficacy in NSCLC.**

Anaís Sánchez-Castillo^1^, Elien Heylen^2^, Judith Hounjet^1^, Kim G. Savelkouls^1^, Natasja G. Lieuwes^3^, Rianne Biemans^3^, Ludwig J. Dubois^3^, Kobe Reynders^1,4^, Kasper M. Rouschop^1^, Rianne D.W. Vaes^1^, Kim De Keersmaecker^2^, Maarten Lambrecht^5^, Lizza E.L. Hendriks^6^, Dirk K.M. De Ruysscher^1^, Marc Vooijs^1^, Kim R. Kampen^1,2^

^1^ Department of Radiation Oncology (MAASTRO), GROW School for Oncology and Reproduction, Maastricht University Medical Center+, Maastricht, The Netherlands

^2^ Department of Oncology, Laboratory for Disease Mechanisms in Cancer, KU Leuven, and Leuven Cancer Institute (LKI), Herestraat 49, 3000 Leuven, Belgium

^3^ Department of Precision Medicine, The M-Lab, GROW School for Oncology and Reproduction, Maastricht University, Maastricht, The Netherlands

^4^ Department of Oncology, Experimental Radiation Oncology, KU Leuven, and Leuven Cancer Institute (LKI), Herestraat 49, 3000 Leuven, Belgium

^5^ Department of Radiation Oncology, University Hospital Leuven, Leuven, Belgium

^6^ Department of Pulmonology, GROW School for Oncology and Reproduction, Maastricht University Medical Center+, Maastricht, The Netherlands

Corresponding author: Kim R. Kampen, Department of Radiation Oncology (MAASTRO), GROW School for Oncology and Reproduction, Maastricht University Medical Center+, Maastricht, The Netherlands, k.kampen@maastrichtuniversity.nl, tel. +32 (0)4794793298.

Disclosures

The authors declare no potential conflicts of interest.

Supplementary material and methods

**Cell Cycle assay**

Cell cycle analysis was performed using Propidium Iodide (PI) staining. Cells were seeded and allowed to attach overnight. Next, cells were treated with sertraline for 48h and irradiated. After 24 hours, cells were fixated with methanol and incubated with 1 µg/ml of PI, 100 µg/ml of RNAse A and 0.1% TritonX-100 in PBS for 30 min at RT. An unstained sample was used for compensation and correction for background and auto-fluorescence. The flow cytometry results were analyzed with FlowJo™ Software v10.8 using the Watson Pragmatic algorithm (61).

**Flow cytometry**

Cells were seeded at their respective optimal seeding densities and allowed to attach overnight. Next, cells were treated with sertraline for 48 hours and subsequently irradiated. After 24 hours, NSCLC were collected and stained with the Alexa Fluor® 647conjugated Phospho-Histone H2A.X (Ser139) antibody (Cell Signaling) or Pacific Blue conjugated cleaved Caspase-3 (Asp175) antibody (cell signaling) after fixation and permeabilization with 100% methanol.

**Western Blot**

Cells were seeded at their respective optimal seeding densities and allowed to attach overnight. Next, cells were treated with sertraline for 48 hours and subsequently irradiated. After 24 hours, cells were lysed using RIPA buffer (1M sodium orthovanadate and 1M NaF) and cell lysates were sonicated and centrifuged to recover the supernatant. Protein concentration was determined using Bio-Rad Protein Assay. Proteins were boiled with laemmli sample buffer (Biorad) plus 2-mercaptoethanol as a loading buffer. Equal amounts of extracts were loaded in 4–15% Criterion TGX Precast Midi Protein gels (Biorad). Proteins were transferred to PVDF membranes (Thermofisher) using a Power Blotter–Semi-dry Transfer System (Thermofisher) and incubated overnight with p21 Waf1/Cip1 (12D1) Rabbit (#2947, Cell Signaling) and Phospho-ATM (Ser1981) (D25E5) Rabbit (13050, Cell Signaling) antibodies diluted in 5% skimmed milk in Tris-Buffered NaCl Solution with Tween 20 (TBST). After washing with TBST, membranes were incubated for 1 h at room temperature with the secondary antibodies conjugated to horseradish peroxidase (HRP) anti-rabbit IgG (Cell Signaling) or anti-mouse IgG (Cell Signaling). ECL reagents (Sigma-Aldrich) were used to visualize the proteins.

**BrdU-incorporation staining**

Cells were seeded and allowed to attach overnight. Next, cells were treated with 10μM sertraline for 48 h and subsequently irradiated. After 24h, BrdU (10μM) was added to the culture medium for 1 h, after which cells were fixed in 4% paraformaldehyde for 20 min. After permeabilization with 0.1% triton X-100 for 20 min, the cells were treated with 2 M HCl for 30 min at 37°C and twice with 0.1 M borate for 5 min. After blocking in 10% FCS for 30 min, cells were incubated with primary antibody (anti-BrdU, AbD serotec, OBT0030S) 1/50 for 90 min and with secondary antibody (FITC-Goat Anti-Rat IgG, Invitrogen, 62-9511) 1/200 for 1 h. Nuclei were stained with Hoechst for 10 min. The amount of (BrdU-positive) nuclei was quantified using Image J software and cell proliferation was expressed as the ratio of BrdU positive nuclei from the total amount of nuclei.

   Supplementary figures

**
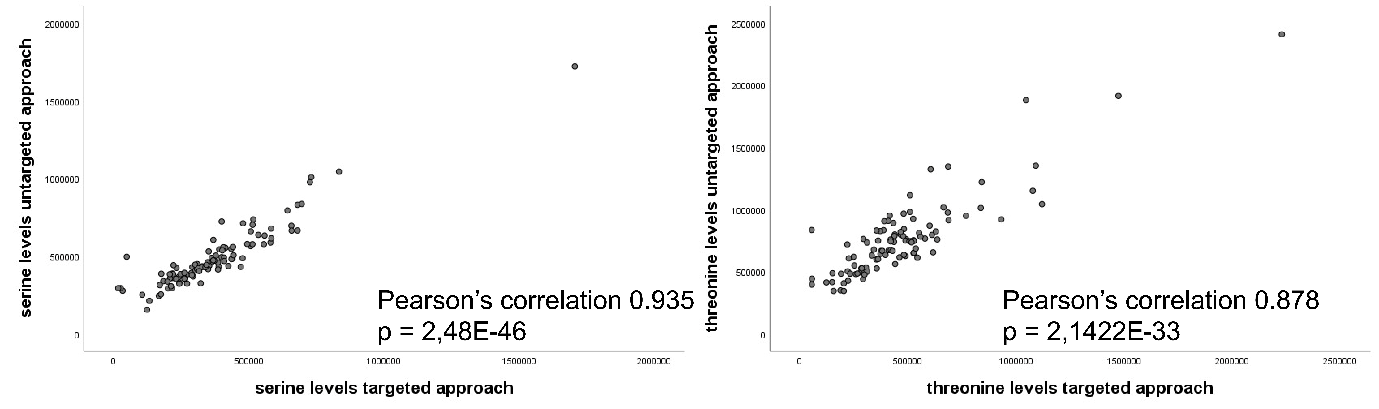
**

**Figure S1. Reproducibility of plasma metabolites by mass spactrometry analysis.** The plasma samples from NSCLC patients were remeasured after a few weeks to reassure reproducibility of metabolites. Strong correlatons and reproducibility was observed.


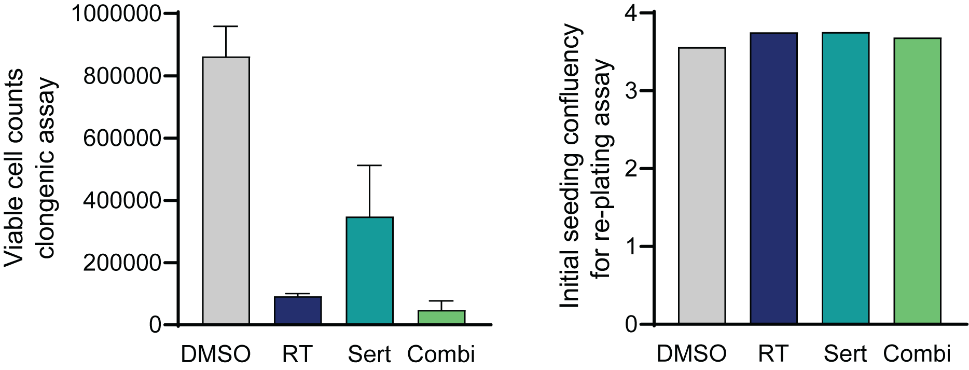


**Figure S2. Calu-6 clonogenic and replate.** Left; Calu-6 absolute viable cell counts after clonogenic assay. Right; Calu-6 seeding confluence check after replating 500 cells/well for accuracy using incucyte.


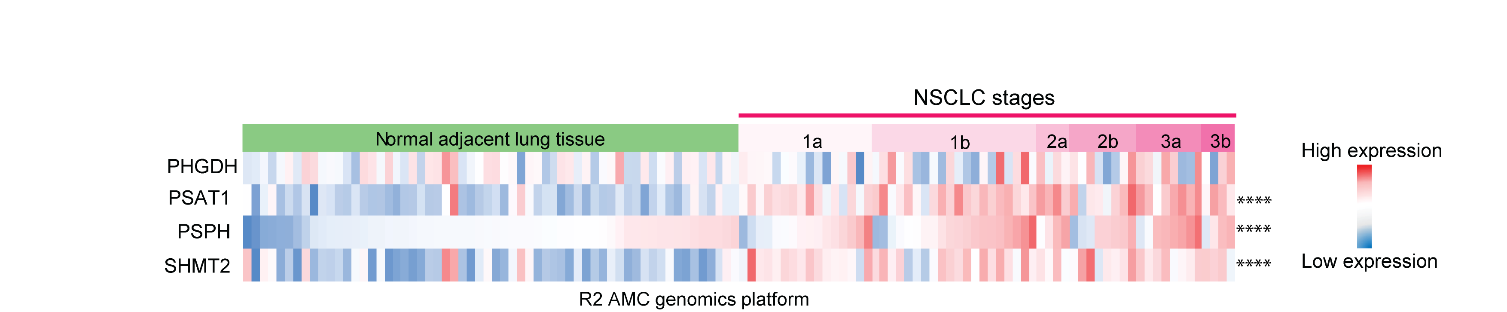


**Figure S3. Overexpression of ser/gly synthesis enzymes expression in NSCLC compared to normal adjacent lung tissue from paired patients.** Heatmap used to visualize the expression of ser/gly synthesis enzymes, i.e. PHGDH, PSAT1, PSPH and SHMT2 by RNA-Seq in NSCLC patient tumors compared to the paired normal adjacent lung tissue. The log2 transformed data was collected from R2 AMC genomics platform using the publicly available (GSE19804) dataset. Fisher’s exact test was used to calculate the frequency of samples with high expression in NSCLC or normal paired tissue samples, calculated as median log2 expression normal +0.4. ****p-value <0.0001.


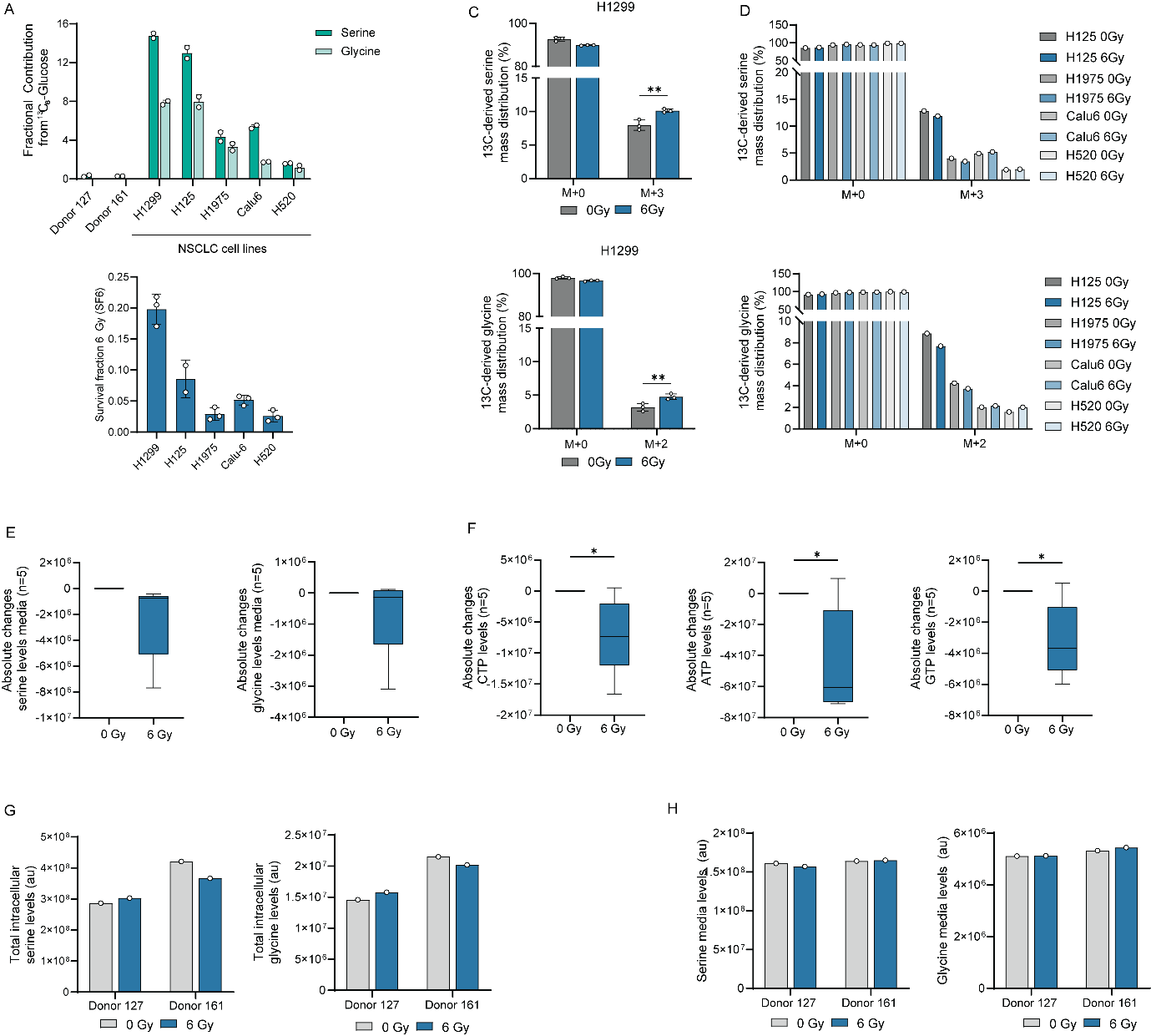


**Figure S4. Radioresistant NSCLC cell lines have higher basal ser/gly synthesis with increased ser/gly synthesis in response to RT.** Serine is synthesised from the glycolytic intermediate 3-PG, producing labelled serine (m+3) and serine can generate labelled glycine (m+2). **A)** Fractional contribution from U-^13^C_6_-glucose for ser/gly synthesis in healthy lung and NSCLC cell models.  **B)** Survival fraction of NSCLC cell lines exposed to 6 Gy irradiation (SF6), normalized to the non-irradited cells for each cell line. **C,D)** ^13^C-derived ser/gly mass distribution in the radioresistant cell line H1299 and different NSCLC cell lines, 24 h post-irradiation with 6 Gy and non-irradiated controls. A two-way ANOVA with Šídák's multiple comparisons test has been performed, where **p-value < 0.01. **E)** Absolute intracellular changes of serine and glycine levels in the media of different NSCLC cell models, 24 h post-irradiation with 6 Gy and non-irradiated controls. **F)** Absolute intracellular changes of CTP, ATP and GTP in the different NSCLC cell models, 24 h after exposure of 6 Gy irradiation. **G**, **H)** Serine and glycine levels in cells and media in two healthy lung donors, i.e. lung donor 127 and 161, 24 h after exposure of 6 Gy irradiation and their respective non-irradiated controls. Data are represented as mean ± standard deviation. Each dot in the graphs represent an independent biological replicate.


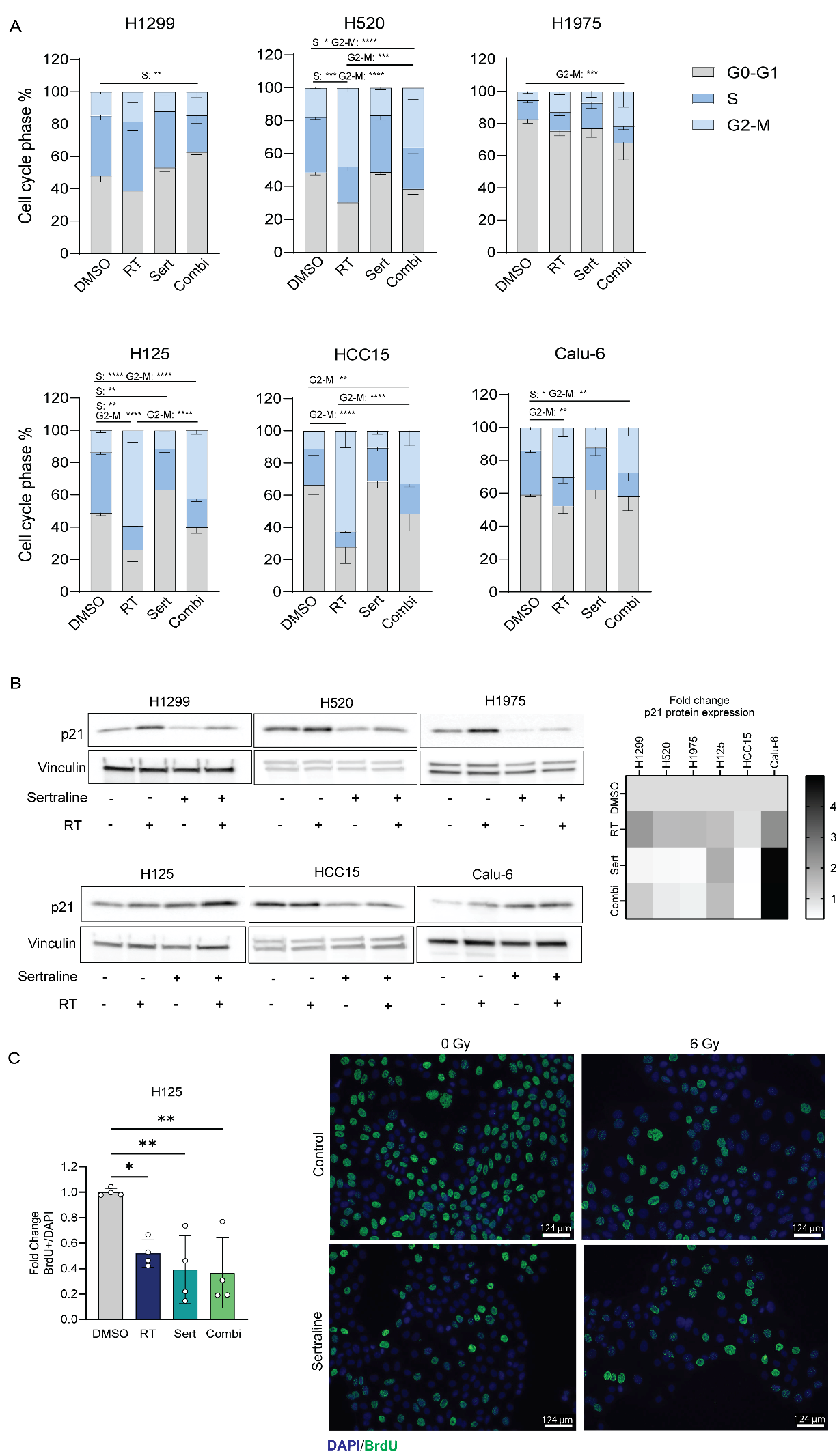


**Figure S5. The treatment of NSCLC cell lines with RT, sertraline and the combination treatment affect cell cycle progression. A)** Graphs showing % of cells in the different cell cycle phases in NSCLC cell lines after exposure to RT, sertraline and the combination treatment. NSCLC cell lines were pre-treated with sertraline for 48h before irradiation. The analysis of the cell cycle phases was performed 24h post-RT. The graphs show the % of cells in the different cell cycle phases analyzed by flow cytometry using the Watson Pragmatic algorithm. A Two-way ANOVA statistical test was used with a Tukey’s post hoc test for intergroup comparisons. **B)** Expression of p21 protein by western blot in the different NSCLC cell lines after exposure to sertraline, RT, and the combination treatment. Heat map showing the fold change of p21 protein expression, normalized to DMSO condition. **C)** Graph showing fold change of the ratio of BrdU+ and the total number of H125 cells after RT, sertraline, and the combination treatment. H125 cells were incubated for 1 hour with BrdU, 24h post-RT, before performing the staining. Representative images of BrdU staining of H125 cell line upon the different treatments, in blue DAPI positive cells and in green the BrdU positive cells. Data are represented as mean ± standard deviation. Individual dots represent independent observations. One-way ANOVA statistical test was used for BrdU analysis with Tukey’s post hoc test for intergroup comparisons. Statistical analysis *p-value < 0.05, **p-value < 0.01, ***p-value < 0.001, ****p-value <0.0001.


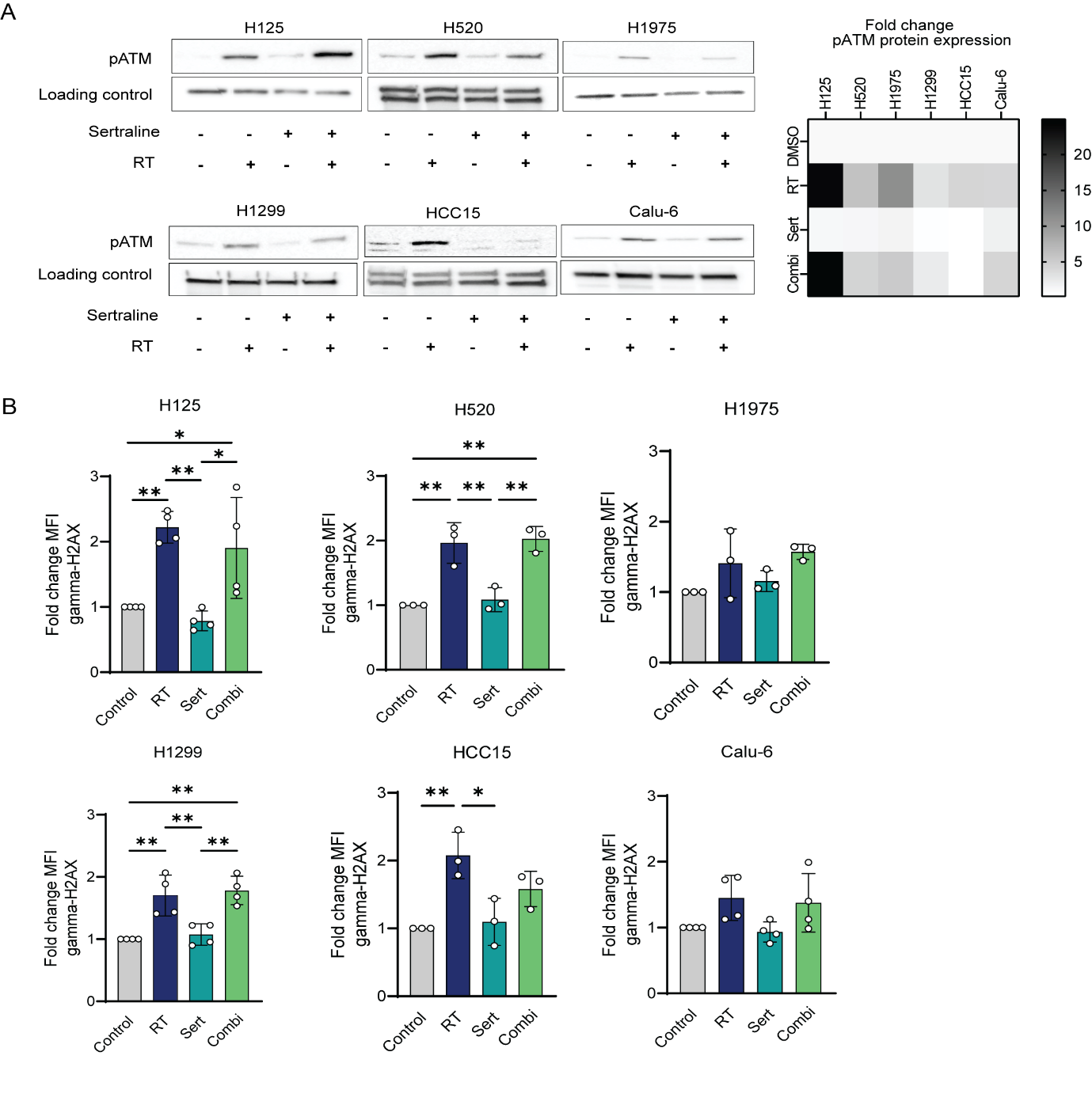


**Figure S6. DNA Damage response in NSCLC cell lines after exposure to RT and the combination treatment. A)** p-ATM protein expression by western blot in the different NSCLC cell lines after exposure to sertraline, RT, and the combination treatment, 24 h post-RT. As loading controls, lamin was used for H125, tubulin for H1975 cell line, and vinculin was used for H520, H1299, HCC15 and Calu-6 cell lines. Heat map showing the fold change of pATM protein expression, normalized to DMSO condition. **B)** Gamma-H2AX expression detected by flow cytometry in NSCLC cell lines after treatment with sertraline, RT and the combination treatment, 24 h post-RT. The graphs show the fold change of median fluorescent intensity (MFI) of n≥3 independent experiments. One-way ANOVA statistical test was used with Tukey’s post hoc test for intergroup comparisons. Statistical analysis *p-value < 0.05, **p-value < 0.01.

**
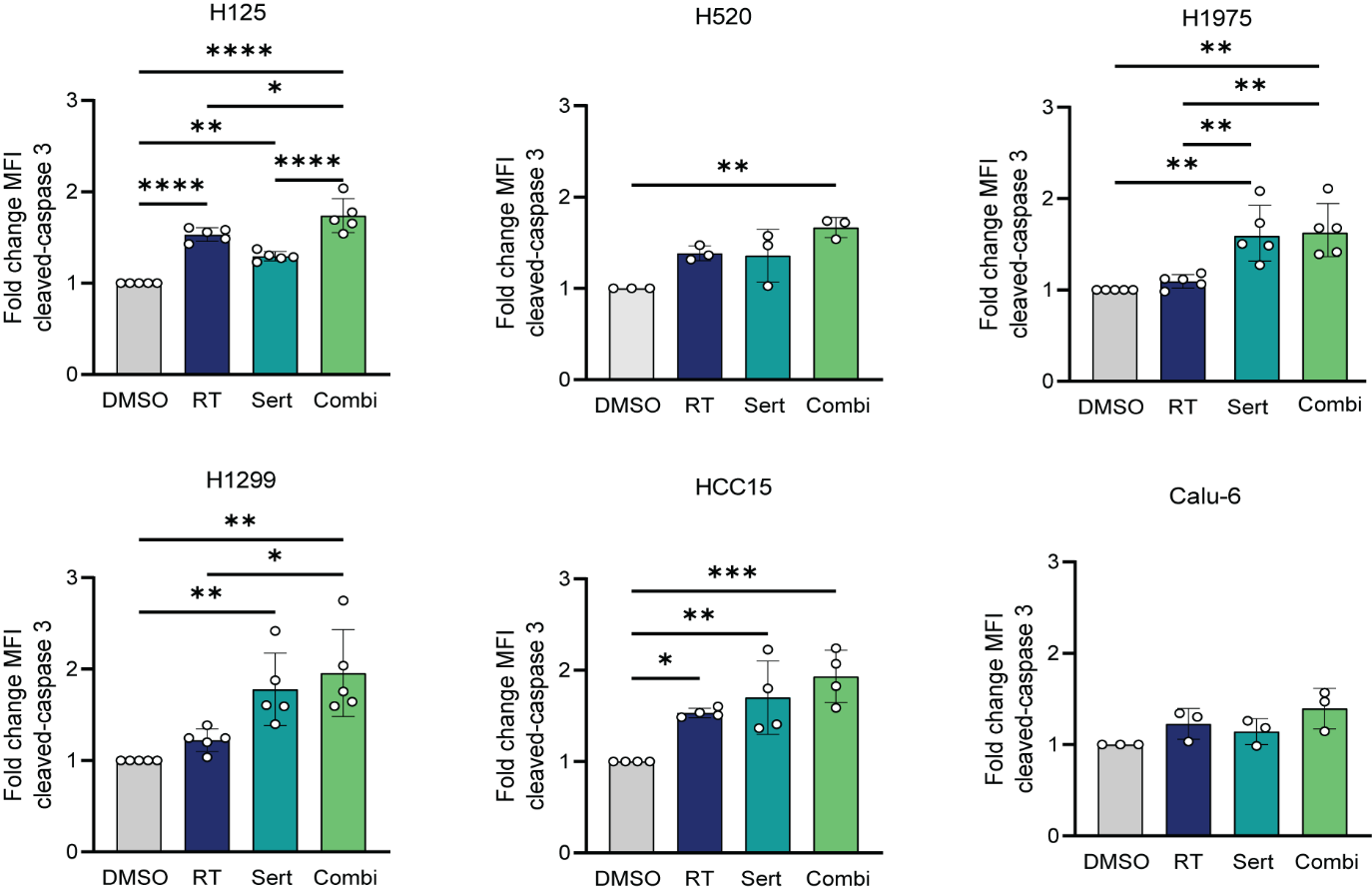
**

**Figure S7. Sertraline induces apoptosis in NSCLC cell lines.** Cleaved-caspase 3 expression detected by flow cytometry in NSCLC cell lines after treatment with sertraline and 24h post-RT. The graphs show the fold change of mean fluorescent intensitiy (MFI) of n≥3 independent experiments. One-way ANOVA statistical test was used with Tukey’s post hoc test for intergroup comparisons. Statistical analysis *p-value < 0.05, **p-value < 0.01, ***p-value < 0.001, ****p-value <0.0001.

**
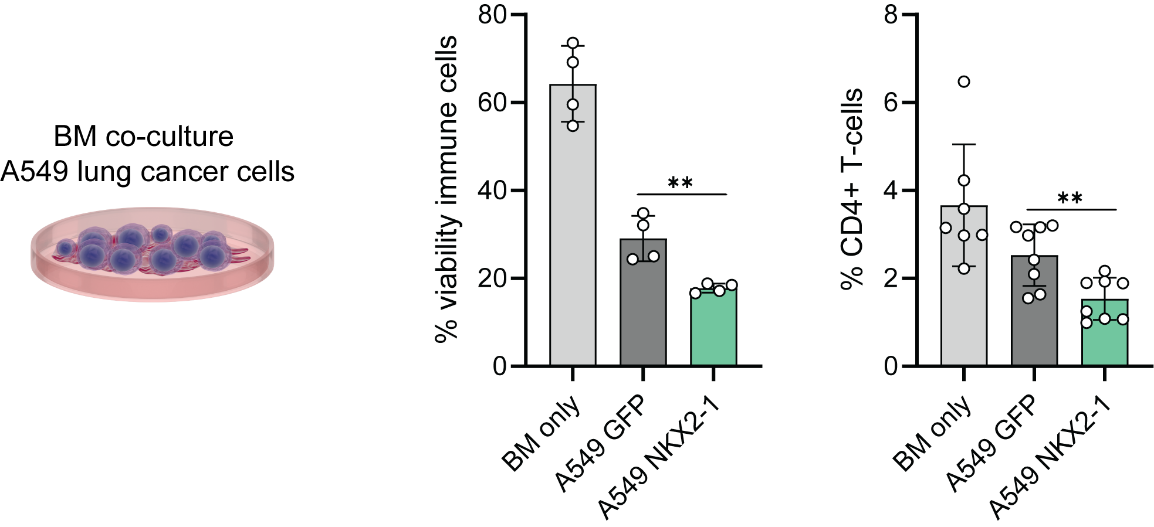
**

**Figure S8. Ser/Gly driven NSCLC suppression of immune cells *ex vivo* using the isogenic NKX2-1 A549 model.** Co-culture experiment with isogenic A459 GFP control and NKX.2-1 overexpression A549 cells together with mouse bone marrow (BM) cells (C57BL/6). Graphs show the percentage (%) of viable immune cells and CD4+ T cells, using n=2 donor mice and n=2 biological replicates for the percentage (%) of viable immune cells, and additionally n=2 technical replicates for the percentage (%) of CD4+ T cells. Unpaired 2-tailed t-test was used. Statistical analysis **p-value < 0.01.


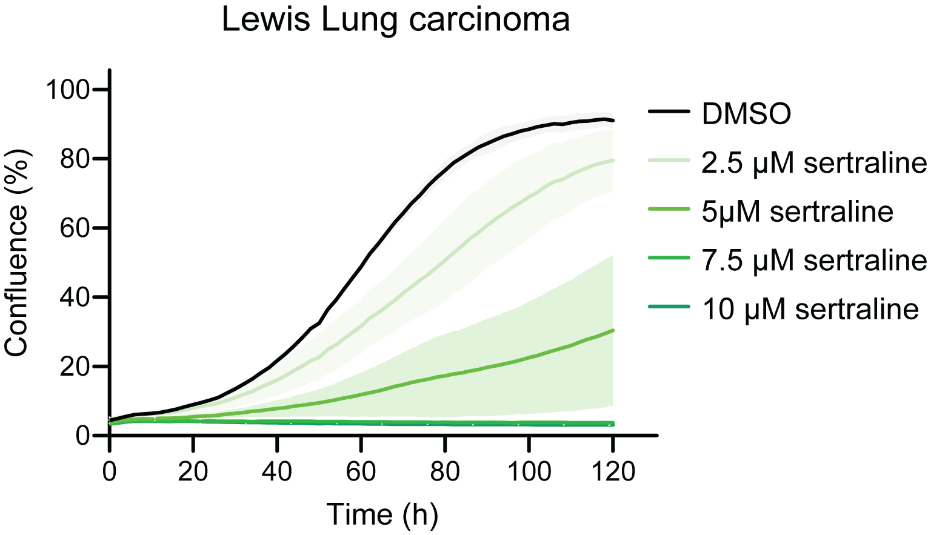


**Figure S9. Lewis Lung carcinoma (LLC) cells are sensitive to sertraline treatment.** Growth curves of LLC cell line treated with different concentrations of sertraline and using DMSO as control. Data are presented as mean ± SD of 6 replicate wells and are representative of n≥3 independent experiments.

**
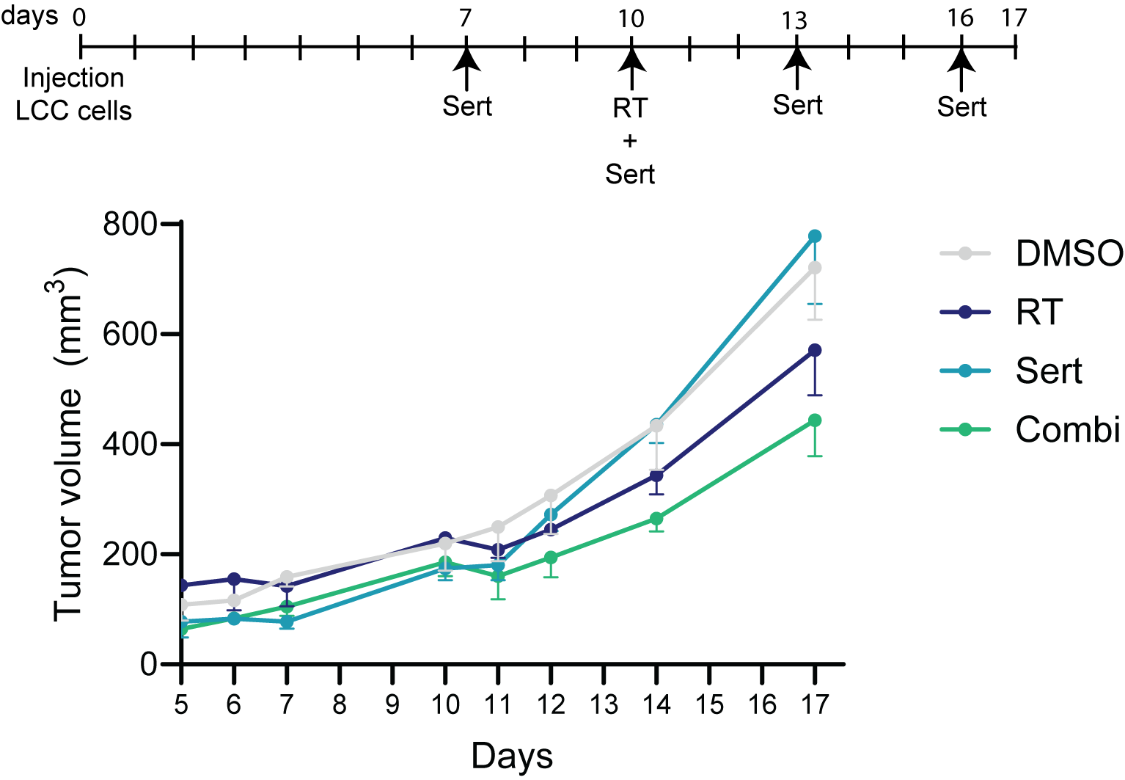
**

**Figure S10. The combination treatment reduces LLC tumor growth *in vivo*.** Schematic representation of treatment schedule and graph showing tumor growth as tumor volume (mm^3^) in the different treatment groups: DMSO, RT 15 Gy, sertraline 15mg/kg, and combination. Data are presented as mean ± SEM of n≥3 mice in each group.

**
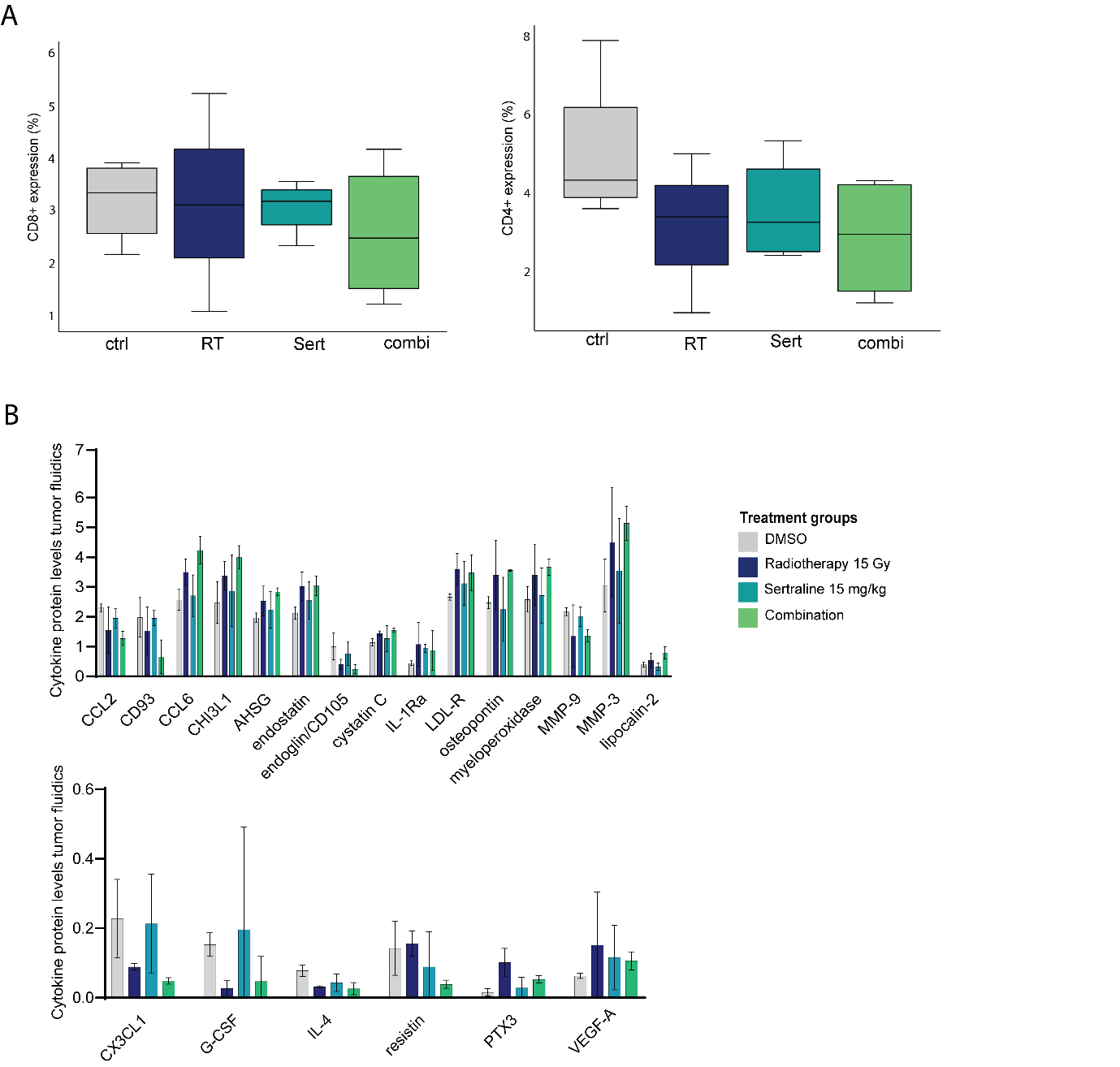
**

**Figure S11. *In vivo* analysis of sertraline and RT combination treatment in tumor growth and influence in the immune tumor microenvironment. A)** Analysis of CD4+ and CD8+ expression by flow cytometry in the dissociated lung tumors for different treatment groups. **B)** Graphs showing the proteins levels of the cytokines that were detected by a cytokine array (proteome profiler mouse XL cytokine array). Data are presented as the mean ±SD for bar graphs.

**
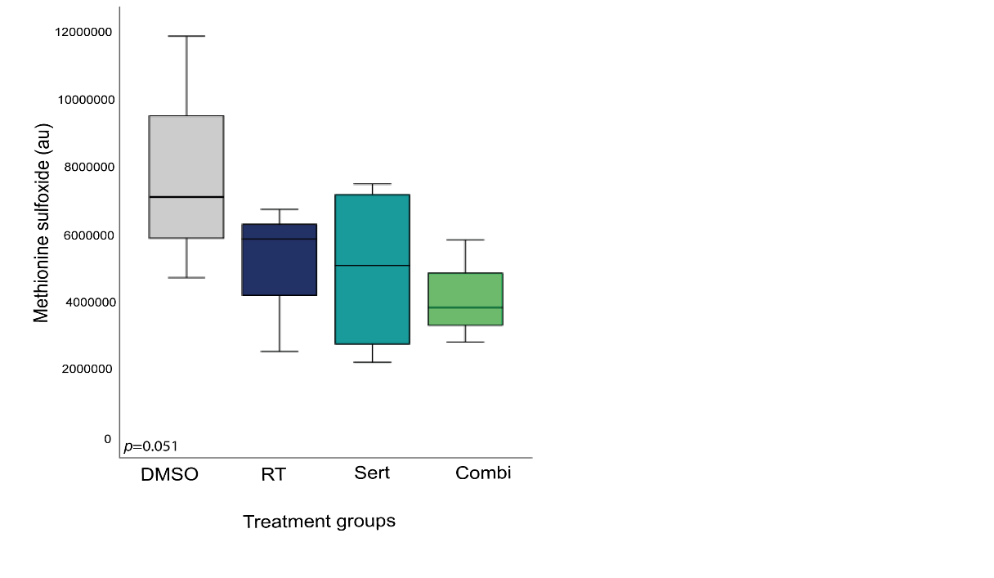
**

**Figure S12. The combination treatment of sertraline and RT reduces methionine sulfoxide serum levels *in vivo.*** Box plots showing the serum levels of methionine sulfoxide in mice treated with DMSO, RT, sertraline as monotherapies and the combination of sertraline and RT. A Jonckheere-Terpstra test was performed, *p*=0.051.

**
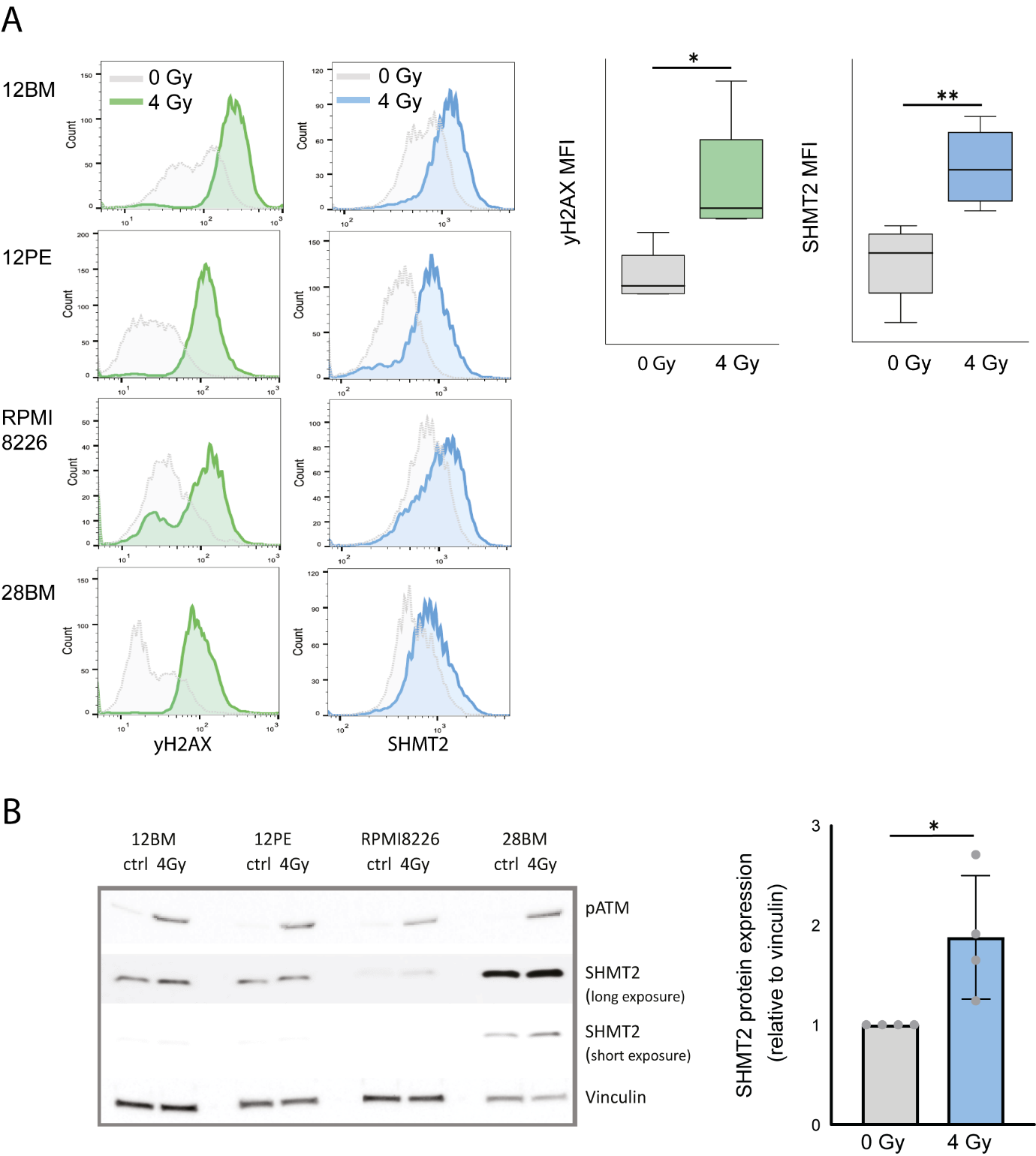
**

**Figure S13. Multiple myeloma cell lines increase expression of SHMT2 in response to irradiation.** Cell lines were obtained from DSMZ and JJC and cultured in RPMI. **A)** Flow cytometry graphs showing the fluorescence intensity of γ-H2AX and SHMT2 in a panel of different multiple myeloma cell lines, 24h post-irradiation with 4 Gy. The graphs represent the mean fluorescence intensity (MFI) values. Unpaired 2-tailed t-test was used. **B)** p-ATM and SHMT2 protein expression by western blot in the different multiple myeloma cell lines 24h after exposure to 4 Gy irradiation. The graphs represent the fold change of SHMT2 protein expression, relative to the loading control vinculin, and normalized to 0 Gy. Unpaired 2-tailed t-test was used. Statistical analysis *p-value < 0.05, **p-value < 0.01.
